# Supplementary material for: AAV-Delivered RNAi Targeting Mutant LDB3 Prevents and Reverses Myofibrillar Myopathy through Mechanosignaling Restoration
Source: bioRxiv. 2026 Mar 31:2026.03.28.715031. Preprint. [Version 1] doi: 10.64898/2026.03.28.715031 (PMC13317614; doi:10.64898/2026.03.28.715031)
Supplement: Supplement 1 [file media-1.pdf]

Supplemental Information

Supplemental figures and legends

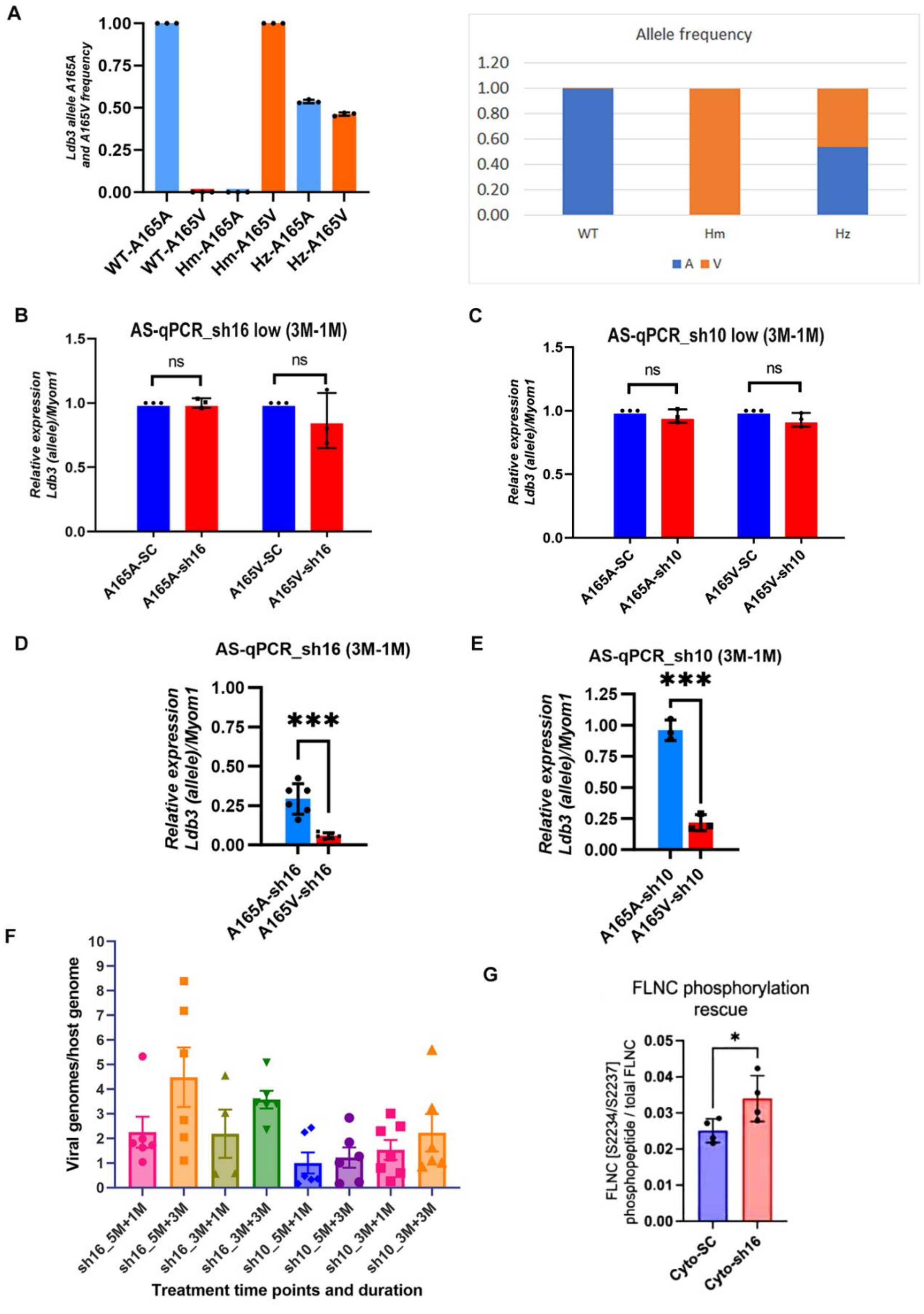

Figure S1. Allele-specific qPCR optimization, AAV therapeutic dose determination, AAV transduction efficiency, and FLNc phosphorylation following AAV9-shRNAmiR treatment.

(A) Validation of allele-specific RT-qPCR using cDNA from WT, *Ldb3*$^{Ala165Val/Ala165Val}$ (Homozygous), and *Ldb3*$^{Ala165Val/+}$ (Heterozygous) mice confirms assay specificity for WT-A165A and A165V alleles.

Three-month-old *Ldb3*$^{Ala165Val/+}$ mice received a single intramuscular injection of sh10 or sh16 into the TA muscle; contralateral TA muscles received scramble control (SC). (B and C) AS-qPCR analysis showing relative expression of WT (A165A) and mutant *Ldb3*-A165V transcripts at one-month post-injection following low-dose AAV9-shRNAmiR (5×10^{11} vg/kg) compared with SC-treated controls in sh16 and sh10 treatment groups (n = 3 mice per group). (D and E) AS-qPCR analysis showing relative expression of WT (A165A) and mutant *Ldb3*-A165V transcripts at one-month post-injection following high-dose AAV9-shRNAmiR (5×10^{12} vg/kg) compared with SC-treated controls in sh16 and sh10 treatment groups (n = 3–6 mice per group). (F) AAV vector genome copies per host genome across treatment groups in sh16- and sh10-treated *Ldb3*$^{Ala165Val/+}$ mice at advanced stage (5 months) and early stage (3 months), analyzed at 1 and 3 months post-injection to assess transduction efficiency (n = 4-6 mice per group). (G) Phosphoproteomic fold-change data presented as a bar-scatter plot showing increased phospho-FLNc (S2234/S2237)/total FLNc ratio following sh16 treatment (3-month-old mice, one-month post-injection) compared with SC (n = 4 mice per group). Student's t test: $p = 0.0457$. Data are presented as mean ± SD (A-G). Statistical significance was determined using Welch's t-Test (B and C) and unpaired two-tailed Student's t tests (D, E and G) (*$P < 0.05$ and ***$P < 0.001$), ns = not significant.

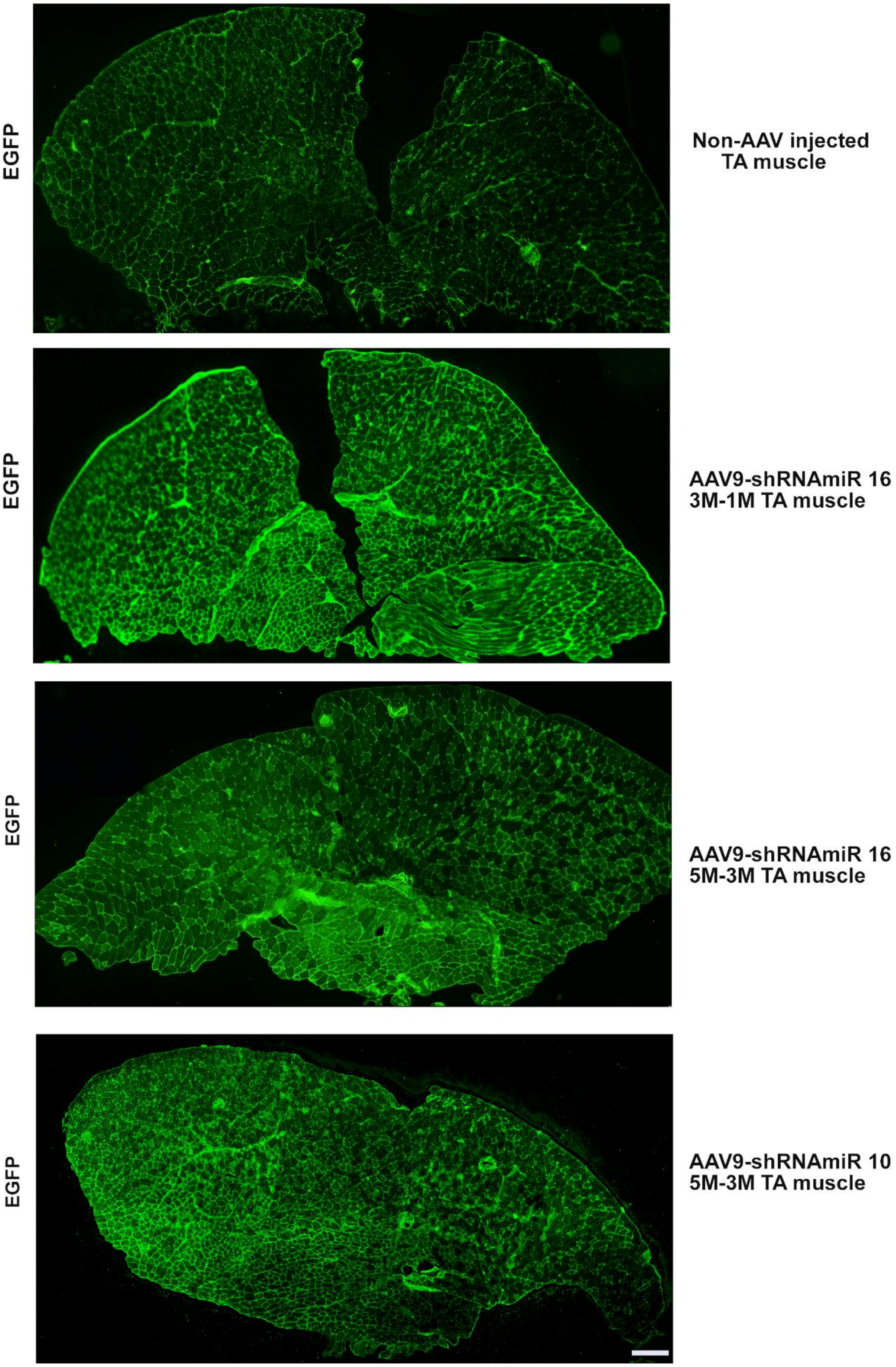
EGFP
Non-AAV injected TA muscle
EGFP
AAV9-shRNAmiR 16 3M-1M TA muscle
EGFP
AAV9-shRNAmiR 16 5M-3M TA muscle
EGFP
AAV9-shRNAmiR 10 5M-3M TA muscle

Figure S2: Efficient skeletal muscle transduction following AAV9-shRNAmiR administration.

Representative IF images of EGFP expression in 8-µm transverse sections of TA muscle from *Ldb3*$^{Ala165Val/+}$ mice following AAV9-shRNAmiR delivery. The top panel shows non-AAV-injected TA muscle stained for EGFP (negative control). Subsequent panels show EGFP expression in TA muscles at 1-month post-injection in 3-month-old mice (3M-1M; age 4 months) and at 3 months post-injection in 5-month-old mice (5M-3M; age 8 months) treated with AAV9-shRNAmiR16 or AAV9-shRNAmiR10. Broad and homogeneous EGFP expression throughout the transverse section demonstrates efficient AAV-mediated skeletal muscle transduction in both early-stage (3M-1M and 3M-3M) and advanced-stage (5M-1M and 5M-3M) treatment paradigms. Scale bar: 250 µm.

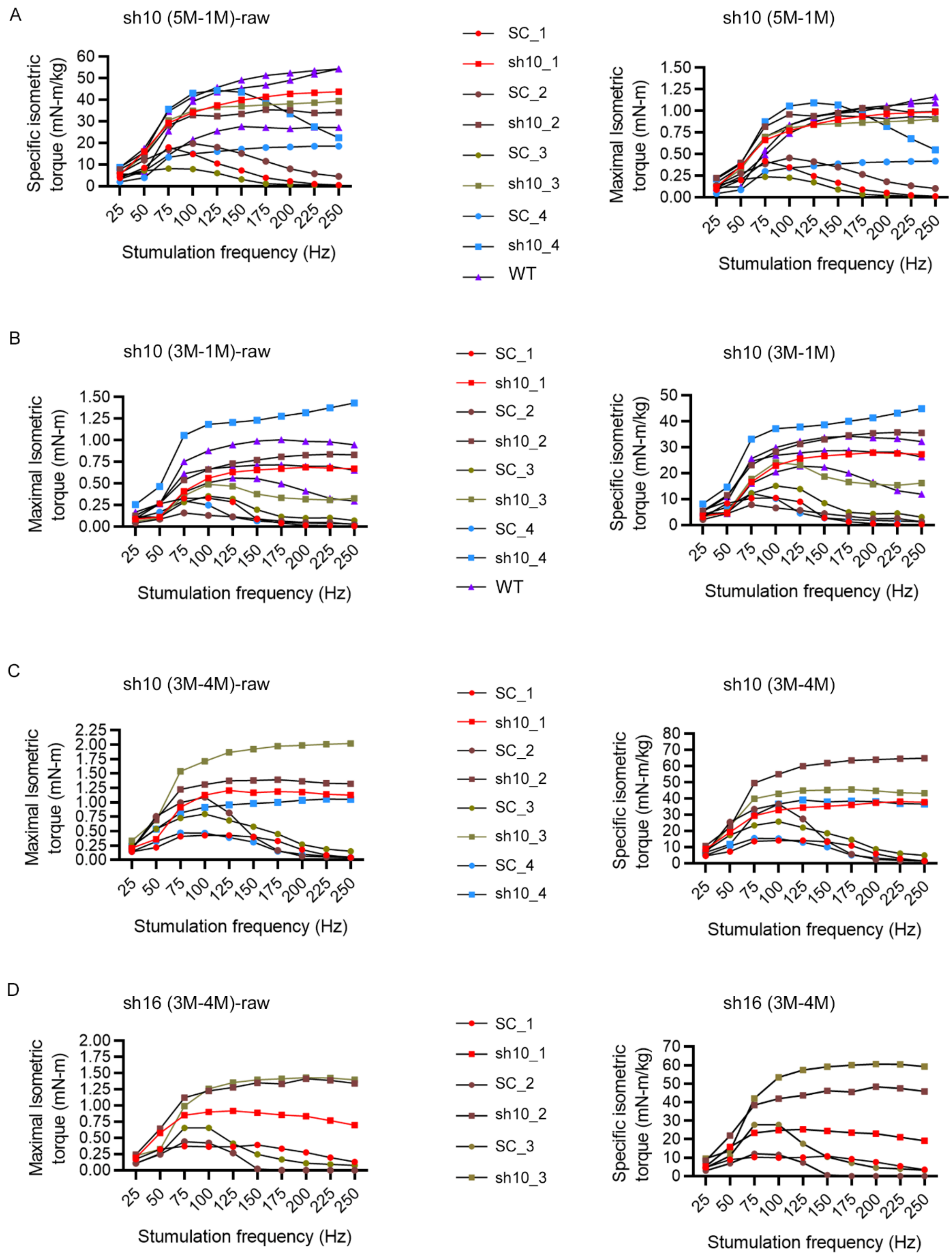
A
sh10 (5M-1M)-raw
Specific isometric torque (mN-m/kg)
Stumulation frequency (Hz)
SC_1
sh10_1
SC_2
sh10_2
SC_3
sh10_3
SC_4
sh10_4
WT
sh10 (5M-1M)
Maximal Isometric torque (mN-m)
Stumulation frequency (Hz)
B
sh10 (3M-1M)-raw
Maximal Isometric torque (mN-m)
Stumulation frequency (Hz)
SC_1
sh10_1
SC_2
sh10_2
SC_3
sh10_3
SC_4
sh10_4
WT
sh10 (3M-1M)
Specific isometric torque (mN-m/kg)
Stumulation frequency (Hz)
C
sh10 (3M-4M)-raw
Maximal Isometric torque (mN-m)
Stumulation frequency (Hz)
SC_1
sh10_1
SC_2
sh10_2
SC_3
sh10_3
SC_4
sh10_4
sh10 (3M-4M)
Specific isometric torque (mN-m/kg)
Stumulation frequency (Hz)
D
sh16 (3M-4M)-raw
Maximal Isometric torque (mN-m)
Stumulation frequency (Hz)
SC_1
sh10_1
SC_2
sh10_2
SC_3
sh10_3
sh16 (3M-4M)
Specific isometric torque (mN-m/kg)
Stumulation frequency (Hz)

Figure S3. Force frequency relationship data from 25 to 250 Hz for each treatment group in *Ldb3*$^{Ala165Val/+}$ and WT-TA muscle.

Force-frequency relationships showing raw maximal isometric force (left) and body-weight–normalized specific isometric torque (right) generated by TA muscles in response to increasing stimulation frequencies. (A) sh10 treatment (5M-1M), (B) sh10 treatment (3M-1M), (C) sh10 treatment (3M-4M) and (D) sh16 treatment (3M-4M). Within each panel, shRNAmiR-treated TA muscles are compared with contralateral scramble control (SC)-treated muscles. PBS-treated WT mice are included where indicated (n = 3–4 per group) and serve as a reference for the sh10 and sh16 (3M–4M) treatment groups shown in the main figures.

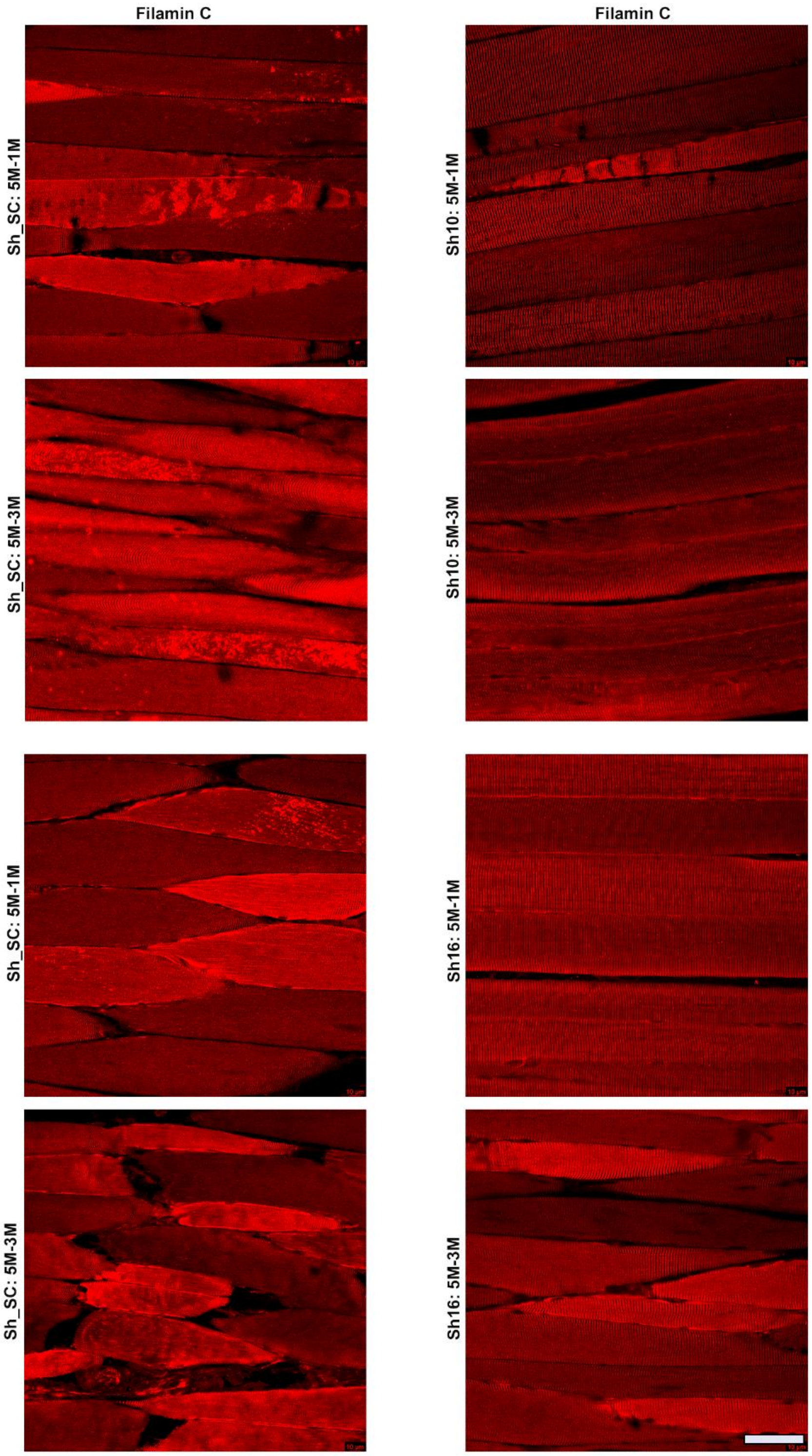

Figure S4. Therapeutic *Ldb3* suppression reduces filamin C aggregation in advanced-stage *Ldb3*$^{Ala165Val/+}$ mice.

Representative IF images of filamin C in PFA-perfused longitudinal TA muscle sections from advanced-stage (5-month-old) *Ldb3*$^{Ala165Val/+}$ mice treated with sh-SC, sh10, or sh16 and analyzed at 1 month (5M-1M; age 6 months) or 3 months (5M-3M; age 8 months) post-injection (n = 4-6 per group).

Left panels (sh-SC) show extensive filamin C aggregation with progressive Z-disc disorganization at 5M-1M and marked Z-disc disruption at 5M-3M. Right panels show the corresponding sh10- (upper rows) and sh16-treated (lower rows) muscles at both time points, demonstrating marked reduction of filamin C aggregates and restoration of Z-disc architecture relative to SC-treated controls. Scale bars represent 50 μm.

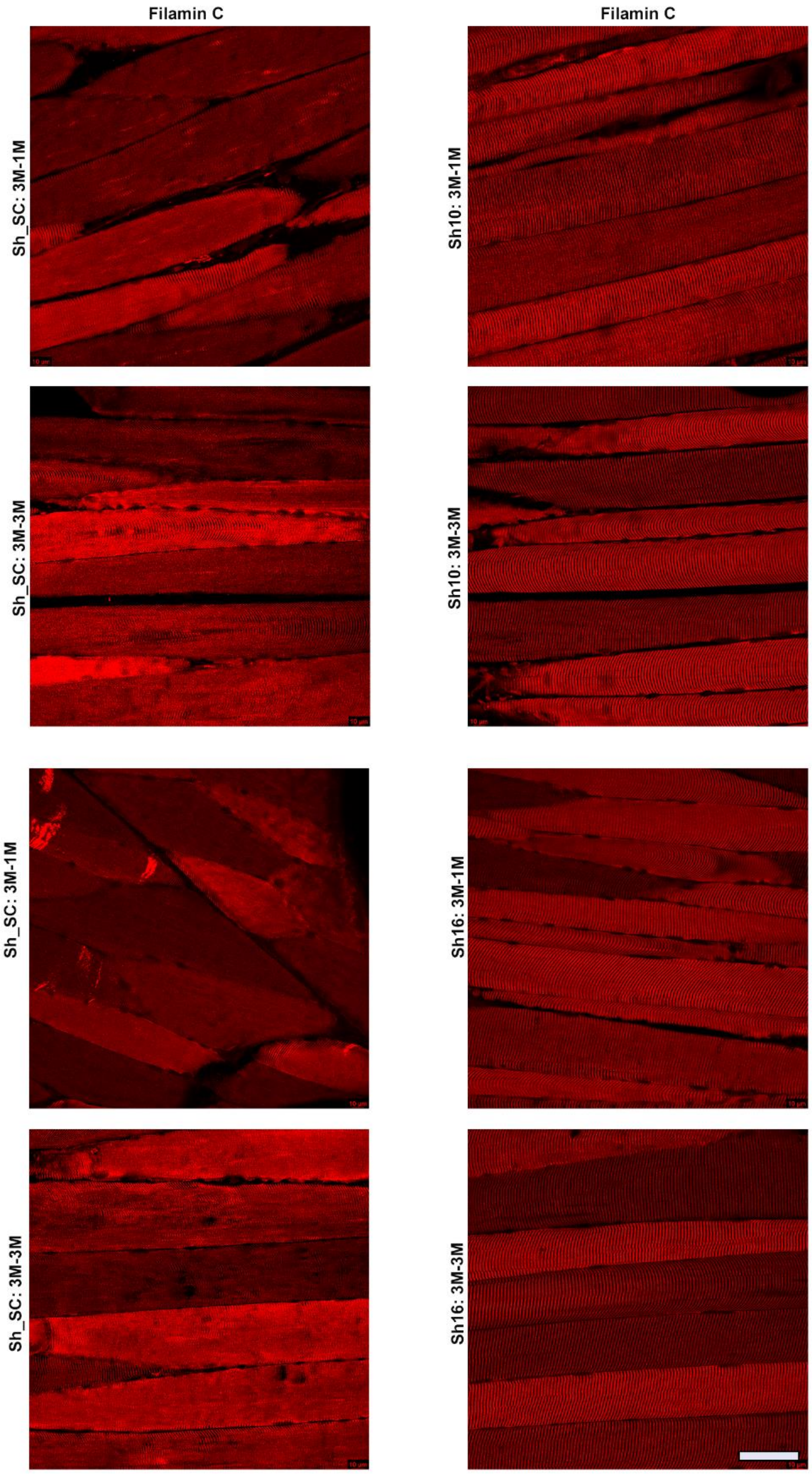

Figure S5: Early-stage mutant *Ldb3* suppression prevents filamin C aggregation and Z-disc disruption in *Ldb3*$^{Ala165Val/+}$ mice.

Representative IF images of filamin C in PFA-perfused longitudinal TA muscle sections from early-stage (3-month-old) *Ldb3*$^{Ala165Val/+}$ mice treated with sh-SC, sh10, or sh16 and analyzed at 1 month (3M-1M; age 4 months) or 3 months (3M-3M; age 6 months) post-injection (n = 4-6 per group). Left panels (sh-SC) show the onset of filamin C aggregation at 3M-1M, with aggregates extending across multiple Z-discs, and increased aggregation with evident Z-disc disorganization at 3M-3M. Right panels show the corresponding sh10- (upper rows) and sh16-treated (lower rows) muscles at both time points, in which filamin C aggregation is absent or markedly reduced and Z-disc architecture remains preserved. Scale bars represent 50 μm.

Supplemental tables

Table S1: siRNA design and shRNA sequence BLAST analysis

Tab 1. siRNA scoring based on algorithmic prediction

Tab 2. Complete BLAST output for shRNA sequence homology.

Tab 3. Top predicted off-target matches identified by shRNA sequence BLAST analysis.

Tab 4. Vector maps

Table S2: TMT-based phosphoproteomic analysis of differential phosphorylation between scrambled control- and LDB3-sh16-treated TA muscle.

Combined cytosolic and myofibrillar phosphosite-level datasets (Tabs 2 and 3) were used to generate the 35 unique significantly altered phosphoproteins listed in Tab 1 for KEGG pathway enrichment analysis, whereas combined cytosolic and myofibrillar datasets (Tabs 4 and 5) were used for KEA3 kinase enrichment analysis.

Tab 1. List of 35 unique significantly altered phosphoproteins with phosphosite details (≥1 significant phosphosite per protein) used for KEGG pathway enrichment analysis.

Tab 2. Cytosolic fraction phosphosite-level dataset, including annotated sequences, modification sites, quantitative values (log_2 means and linear differences), and Welch's t test uncorrected and corrected p values.

Tab 3. Myofibrillar fraction phosphosite-level dataset, including annotated sequences, modification sites, quantitative values (log_2 means and linear differences), and Welch's t test uncorrected and corrected p values.

Tab 4. Significantly regulated cytosolic phosphoproteins used for KEA3 kinase enrichment analysis.

Tab 5. Significantly regulated myofibrillar phosphoproteins used for KEA3 kinase enrichment analysis.

Table S3. PKCα-responsive structural, mechanosensory, and metabolic phosphoproteins identified by TMT-based phosphoproteomic analysis of LDB3-sh16–treated TA muscle

Table S4. Enrichr-KG analysis of proteins with treatment-responsive phosphosites in sh16-treated *Ldb3*$^{Ala165Val/+}$ TA muscle

Table S5. Complete cytosolic fraction phosphosite-level dataset from TMT-based phosphoproteomic analysis of scrambled control– and LDB3-sh16–treated TA muscle.

Table S6. Complete myofibrillar fraction phosphosite-level dataset from TMT-based phosphoproteomic analysis of scrambled control– and LDB3-sh16–treated TA muscle.

siRNA scoring

siRNA	siRNA Sense sequence 5' --> 3'	GC content (%)		#'A/U' at position 15-19		Absence of Internal repeats [Tm (°C)]		Position 19 with 'A'		Position 3 with 'A'		Position 10 with 'U'		A base other than 'G' or 'C' at position 19		A base other than 'G' at position 13		
siRNA	siRNA Sense sequence 5' --> 3'	value	Points	value	points	value	points	y/n[a]	points	y/n[a]	points	y/n[a]	points	y/n[a]	points	y/n[a]	points	Score
1. Acatcctgtgagtacatgc	ACA UCC UGU GAG UAC AUG CUU	47.4	1	16, 17	2	47	1	C	0	A	1	G	0	Y	-1	U	0	3
2. aAcatcctgtgagtacatg	AAC AUC CUG UGA GUA CAU GUU	42.1	1	15,17,18	3	44	1	G	0	C	0	U	1	Y	-1	G	-1	3
**3.** ataAcatcctgtgagtaca	UAA CAU CCU GUG AGU ACA UUU	36.8	1	15,16,18,	4	42	1	U	0	A	1	G	0	N	0	A	0	6
**4.** ataAcatcctgtgagtaca	AUA ACA UCC UGU GAG UAC AUU	33.3	1	16, 17, 19	3	42	1	A	1	A	1	U	1	N	0	G	0	7
**5.** gataAcatcctgtgagta	GAU AAC AUC CUG UGA GUA CUU	38.1	1	15,17,18	3	44	1	C	0	U	0	C	0	Y	-1	U	0	3
6. tgataAcatcctgtgagta	UGA UAA CAU CCU GUG AGU AUU	38.8	1	16,18,19	3	42	1	A	1	A	1	C	0	N	0	G	-1	5
7. atgataAcatcctgtgagt	AUG AUA ACA UCC UGU GAG UUU	38.8	1	15,17,19	3	42	1	U	0	G	0	U	1	N	0	U	0	5
8. catgataAcatcctgtgag	CAU GAU AAC AUC CUG UGA GUU	42.1	1	16,18	2	44	1	G	0	U	0	A	0	Y	-1	C	0	2
9. ccatgataAcatcctgtga	CCA UGA UAA CAU CCU GUG AUU	38.1	1	15, 17, 17	3	44	1	A	1	A	1	C	0	N	0	C	0	6
10. tccatgataAcatcctgtg	UCC AUG AUA ACA UCC UGU GUU	38.1	1	16, 18	2	44	1	G	0	C	0	A	0	Y	-1	U	0	2
11. gtccatgataAcatcctgt	GUC CAU GAU AAC AUC CUG UUU	38.1	1	17, 19	2	44	1	U	0	C	0	A	0	N	0	A	0	3
12. cgtccatgataAcatcctg	CGU CCA UGA UAA CAU CCU GUU	47.4	1	15,18	2	47	1	G	0	U	0	U	1	Y	-1	C	0	3
13. gcgtccatgataAcatcct	GCG UCC AUG AUA ACA UCC UUU	47.4	1	15,16,19	3	47	1	U	0	G	0	A	0	N	0	A	0	4
14. ggcgtccatgataAcatcc	GGC GUC CAU GAU AAC AUC CUU	52.6	1	16,17	2	47	1	C	0	C	0	G	0	Y	-1	A	0	2
15. tggcgtccatgataAcatc	UGG CGU CCA UGA UAA CAU CUU	47.4	1	15,17,18	3	47	1	C	0	G	0	U	1	Y	-1	U	0	4
16. atggcgtccatgataAcat	AUG GCG UCC AUG AUA ACA UUU	38.1	1	15,16,18,	4	44	1	U	0	G	0	A	0	N	0	A	0	5
17. gatggcgtccatgataAca	GAU GGC GUC CAU GAU AAC AUU	47.4	1	15,16,17,	4	47	1	A	1	G	0	C	0	N	0	G	-1	5
18. cgatggcgtccatgataA	CGA UGG CGU CCA UGA UAA CUU	52.6	1	15,16,17,	4	47	1	C	0	U	0	C	0	Y	-1	U	0	4
19. gcgatggcgtccatgata	GCG AUG GCG UCC AUG AUA AUU	52.6	1	16,17,18,	4	47	1	A	1	A	1	U	1	N	0	A	0	8

Criterion	Rule	Scoring
(GC%)	Optimal GC between 36–53%	36–53% = 1; otherwise 0
(#A/U 15–19)	Favor A/U-rich seed flank	1 point per A/U (positions 15–19), max 5
I (Tm)	Avoid strong internal repeats	Tm ≤20 °C = +2; 21–59 °C = +1; ≥60 °C = 0
√ (A19)	Prefer A at position 19	A at 19 = +1
` (A3)	Prefer A at position 3	A at 3 = +1
I (U10)	Prefer U at position 10	U at 10 = +1
II (not G/C19)	Penalize G/C at 19	G or C at 19 = −1
III (not G13)	Penalize G at 13	G at 13 = −1

Homology BLAST: shRNA Sequences

shRNA: {si10-ldb3a165V}
Target Sequence: ACTCACAGGATGTTATCATGGA

Transcripts

PREDICTED: Mus musculus LIM domain binding 3 (Ldb3), transcript variant X4, mRNA	Mus musculus	36.2	36.2	100%	0.21	95.45%	1497	XM_036519019.5
PREDICTED: Mus musculus LIM domain binding 3 (Ldb3), transcript variant X3, mRNA	Mus musculus	36.2	36.2	100%	0.21	95.45%	4728	XM_036519018.5
PREDICTED: Mus musculus LIM domain binding 3 (Ldb3), transcript variant X2, mRNA	Mus musculus	36.2	36.2	100%	0.21	95.45%	4929	XM_036519017.5
Mus musculus LIM domain binding 3 (Ldb3), transcript variant 6, mRNA	Mus musculus	36.2	36.2	100%	0.21	95.45%	1506	NM_001039075.2
Mus musculus LIM domain binding 3 (Ldb3), transcript variant 5, mRNA	Mus musculus	36.2	36.2	100%	0.21	95.45%	4954	NM_001039074.2
Mus musculus LIM domain binding 3 (Ldb3), transcript variant 4, mRNA	Mus musculus	36.2	36.2	100%	0.21	95.45%	4737	NM_001039073.2
Mus musculus LIM domain binding 3 (Ldb3), transcript variant 3, mRNA	Mus musculus	36.2	36.2	100%	0.21	95.45%	4908	NM_001039072.2
Mus musculus strain C57BL/6J chromosome 14, GRCm39	Mus musculus	36.2	4722	100%	0.21	95.45%	125139656	NC_000080.7
Mus musculus strain C57BL/6J chromosome 7, GRCm39	Mus musculus	34.2	4370	100%	0.82	100.00%	144995196	NC_000073.7
Mus musculus strain C57BL/6J chromosome 10, GRCm39	Mus musculus	34.2	3980	100%	0.82	95.24%	130530862	NC_000076.7
Mus musculus strain C57BL/6J chromosome 17, GRCm39	Mus musculus	34.2	3630	100%	0.82	100.00%	95294699	NC_000083.7
Mus musculus strain C57BL/6J chromosome 3, GRCm39	Mus musculus	32.2	5337	100%	3.3	100.00%	159745316	NC_000069.7
Mus musculus strain C57BL/6J chromosome 4, GRCm39	Mus musculus	32.2	6355	100%	3.3	100.00%	156860686	NC_000070.7
Mus musculus strain C57BL/6J chromosome 9, GRCm39	Mus musculus	32.2	4906	100%	3.3	100.00%	124359700	NC_000075.7
Mus musculus strain C57BL/6J chromosome X, GRCm39	Mus musculus	32.2	5091	100%	3.3	95.00%	169476592	NC_000086.8
PREDICTED: Mus musculus predicted gene, 38468 (Gm38468), transcript variant X2, ncRNA	Mus musculus	30.2	30.2	86%	13	94.74%	1245	XR_375892.4
PREDICTED: Mus musculus predicted gene, 38468 (Gm38468), transcript variant X1, ncRNA	Mus musculus	30.2	30.2	86%	13	94.74%	1509	XR_004941619.1
Mus musculus strain C57BL/6J chromosome 1, GRCm39	Mus musculus	30.2	7323	100%	13	100.00%	195154279	NC_000067.7
Mus musculus strain C57BL/6J chromosome 2, GRCm39	Mus musculus	30.2	7102	100%	13	100.00%	181755017	NC_000068.8
Mus musculus strain C57BL/6J chromosome 5, GRCm39	Mus musculus	30.2	5851	100%	13	94.74%	151758149	NC_000071.7
Mus musculus strain C57BL/6J chromosome 6, GRCm39	Mus musculus	30.2	5415	100%	13	100.00%	149588044	NC_000072.7
Mus musculus strain C57BL/6J chromosome 8, GRCm39	Mus musculus	30.2	5002	100%	13	100.00%	130127694	NC_000074.7
Mus musculus strain C57BL/6J chromosome 11, GRCm39	Mus musculus	30.2	4329	100%	13	100.00%	121973369	NC_000077.7
Mus musculus strain C57BL/6J chromosome 12, GRCm39	Mus musculus	30.2	4608	100%	13	100.00%	120092757	NC_000078.7
Mus musculus strain C57BL/6J chromosome 16, GRCm39	Mus musculus	30.2	3598	100%	13	94.74%	98008968	NC_000082.7
Mus musculus strain C57BL/6J chromosome 18, GRCm39	Mus musculus	30.2	3998	100%	13	100.00%	90720763	NC_000084.7
PREDICTED: Mus musculus thyroid hormone receptor interactor 11 (Trip11), transcript variant X4, mRNA	Mus musculus	28.2	28.2	63%	51	100.00%	11764	XM_036157159.1
PREDICTED: Mus musculus thyroid hormone receptor interactor 11 (Trip11), transcript variant X3, misc_...	Mus musculus	28.2	28.2	63%	51	100.00%	9708	XR_004937402.1
PREDICTED: Mus musculus thyroid hormone receptor interactor 11 (Trip11), transcript variant X2, mRNA	Mus musculus	28.2	28.2	63%	51	100.00%	9683	XM_011243986.3
PREDICTED: Mus musculus predicted gene, 35002 (Gm35002), ncRNA	Mus musculus	28.2	28.2	63%	51	100.00%	49623	XR_880957.6
PREDICTED: Mus musculus thyroid hormone receptor interactor 11 (Trip11), transcript variant X1, mRNA	Mus musculus	28.2	28.2	63%	51	100.00%	9760	XM_006515382.4
PREDICTED: Mus musculus predicted gene, 19802 (Gm19802), mRNA	Mus musculus	28.2	28.2	63%	51	100.00%	3490	XM_030245429.1
PREDICTED: Mus musculus predicted gene, 51792 (Gm51792), mRNA	Mus musculus	28.2	28.2	63%	51	100.00%	3514	XM_030245427.1
Mus musculus coiled-coil domain containing 97 (Ccdc97), transcript variant 6, non-coding RNA	Mus musculus	28.2	28.2	81%	51	94.44%	2996	NR_160796.1
Mus musculus coiled-coil domain containing 97 (Ccdc97), transcript variant 3, mRNA	Mus musculus	28.2	28.2	81%	51	94.44%	3119	NM_001368382.1
Mus musculus coiled-coil domain containing 97 (Ccdc97), transcript variant 2, mRNA	Mus musculus	28.2	28.2	81%	51	94.44%	3010	NM_001368381.1
Mus musculus coiled-coil domain containing 97 (Ccdc97), transcript variant 4, mRNA	Mus musculus	28.2	28.2	81%	51	94.44%	3264	NM_001368383.1
Mus musculus coiled-coil domain containing 97 (Ccdc97), transcript variant 5, mRNA	Mus musculus	28.2	28.2	81%	51	94.44%	3108	NM_001368384.1
Mus musculus coiled-coil domain containing 97 (Ccdc97), transcript variant 1, mRNA	Mus musculus	28.2	28.2	81%	51	94.44%	2973	NM_028771.3
Mus musculus ATPase, H+ transporting, lysosomal V1 subunit B2 (Atp6v1b2), mRNA	Mus musculus	28.2	28.2	63%	51	100.00%	2742	NM_007509.3
Mus musculus thyroid hormone receptor interactor 11 (Trip11), mRNA	Mus musculus	28.2	28.2	63%	51	100.00%	6448	NM_028446.1
Mus musculus tetratricopeptide repeat domain 30A1 (Ttc30a1), mRNA	Mus musculus	28.2	28.2	63%	51	100.00%	2862	NM_030188.3
Mus musculus tetratricopeptide repeat domain 30B (Ttc30b), mRNA	Mus musculus	28.2	28.2	63%	51	100.00%	2634	NM_028236.1
Mus musculus tetratricopeptide repeat domain 30A2 (Ttc30a2), mRNA	Mus musculus	28.2	28.2	63%	51	100.00%	2008	NM_001081228.1
Mus musculus ras homolog family member J (Rhoj), mRNA	Mus musculus	28.2	28.2	63%	51	100.00%	2358	NM_023275.2
Mus musculus strain C57BL/6J chromosome 13, GRCm39	Mus musculus	28.2	4603	100%	51	100.00%	120883175	NC_000079.7
Mus musculus strain C57BL/6J chromosome 15, GRCm39	Mus musculus	28.2	3343	100%	51	100.00%	104073951	NC_000081.7
Mus musculus strain C57BL/6J chromosome 19, GRCm39	Mus musculus	28.2	2368	100%	51	100.00%	61420004	NC_000085.7
Mus musculus strain C57BL/6J chromosome Y, GRCm39	Mus musculus	28.2	3922	100%	51	100.00%	91455967	NC_000087.8
PREDICTED: Mus musculus early B cell factor 3 (Ebf3), transcript variant X28, mRNA	Mus musculus	26.3	26.3	59%	201	100.00%	4673	XM_036152653.1
PREDICTED: Mus musculus early B cell factor 3 (Ebf3), transcript variant X26, mRNA	Mus musculus	26.3	26.3	59%	201	100.00%	5874	XM_036152651.1
PREDICTED: Mus musculus early B cell factor 3 (Ebf3), transcript variant X25, mRNA	Mus musculus	26.3	26.3	59%	201	100.00%	5901	XM_036152650.1
PREDICTED: Mus musculus predicted gene, 52733 (Gm52733), transcript variant X2, ncRNA	Mus musculus	26.3	26.3	77%	201	94.12%	869	XR_004942033.1
PREDICTED: Mus musculus period circadian clock 3 (Per3), transcript variant X17, misc_RNA	Mus musculus	26.3	26.3	59%	201	100.00%	5923	XR_004941849.1
PREDICTED: Mus musculus period circadian clock 3 (Per3), transcript variant X16, misc_RNA	Mus musculus	26.3	26.3	59%	201	100.00%	8191	XR_004941848.1
PREDICTED: Mus musculus period circadian clock 3 (Per3), transcript variant X14, misc_RNA	Mus musculus	26.3	26.3	59%	201	100.00%	5626	XR_004941846.1
PREDICTED: Mus musculus period circadian clock 3 (Per3), transcript variant X6, mRNA	Mus musculus	26.3	26.3	59%	201	100.00%	6036	XM_036153826.1
PREDICTED: Mus musculus period circadian clock 3 (Per3), transcript variant X5, mRNA	Mus musculus	26.3	26.3	59%	201	100.00%	6039	XM_011250293.4
PREDICTED: Mus musculus Rho GTPase activating protein 21 (Arhgap21), transcript variant X20, mRNA	Mus musculus	26.3	26.3	59%	201	100.00%	18104	XM_017319263.3
PREDICTED: Mus musculus Rho GTPase activating protein 21 (Arhgap21), transcript variant X19, misc_...	Mus musculus	26.3	26.3	59%	201	100.00%	22243	XR_004940733.1
PREDICTED: Mus musculus Rho GTPase activating protein 21 (Arhgap21), transcript variant X18, misc_...	Mus musculus	26.3	26.3	59%	201	100.00%	21126	XR_373946.5
PREDICTED: Mus musculus Rho GTPase activating protein 21 (Arhgap21), transcript variant X17, misc_...	Mus musculus	26.3	26.3	59%	201	100.00%	21129	XR_373945.5
PREDICTED: Mus musculus Rho GTPase activating protein 21 (Arhgap21), transcript variant X16, misc_...	Mus musculus	26.3	26.3	59%	201	100.00%	22017	XR_004940732.1
PREDICTED: Mus musculus Rho GTPase activating protein 21 (Arhgap21), transcript variant X12, mRNA	Mus musculus	26.3	26.3	59%	201	100.00%	7396	XM_006497546.5
PREDICTED: Mus musculus Rho GTPase activating protein 21 (Arhgap21), transcript variant X11, mRNA	Mus musculus	26.3	26.3	59%	201	100.00%	20821	XM_030252086.2
PREDICTED: Mus musculus Rho GTPase activating protein 21 (Arhgap21), transcript variant X10, mRNA	Mus musculus	26.3	26.3	59%	201	100.00%	7088	XM_030252085.2
PREDICTED: Mus musculus Rho GTPase activating protein 21 (Arhgap21), transcript variant X9, mRNA	Mus musculus	26.3	26.3	59%	201	100.00%	7305	XM_030252084.2
PREDICTED: Mus musculus Rho GTPase activating protein 21 (Arhgap21), transcript variant X8, mRNA	Mus musculus	26.3	26.3	59%	201	100.00%	20824	XM_006497544.5
PREDICTED: Mus musculus Rho GTPase activating protein 21 (Arhgap21), transcript variant X7, mRNA	Mus musculus	26.3	26.3	59%	201	100.00%	7200	XM_036162572.1
PREDICTED: Mus musculus Rho GTPase activating protein 21 (Arhgap21), transcript variant X6, mRNA	Mus musculus	26.3	26.3	59%	201	100.00%	7115	XM_006497543.5
PREDICTED: Mus musculus Rho GTPase activating protein 21 (Arhgap21), transcript variant X4, mRNA	Mus musculus	26.3	26.3	59%	201	100.00%	20851	XM_006497542.5
PREDICTED: Mus musculus Rho GTPase activating protein 21 (Arhgap21), transcript variant X3, mRNA	Mus musculus	26.3	26.3	59%	201	100.00%	7211	XM_006497540.5
PREDICTED: Mus musculus Rho GTPase activating protein 21 (Arhgap21), transcript variant X2, mRNA	Mus musculus	26.3	26.3	59%	201	100.00%	7122	XM_006497539.5
PREDICTED: Mus musculus Rho GTPase activating protein 21 (Arhgap21), transcript variant X1, mRNA	Mus musculus	26.3	26.3	59%	201	100.00%	20854	XM_006497538.5
PREDICTED: Mus musculus protein kinase, cGMP-dependent, type I (Prkg1), transcript variant X1, mRNA	Mus musculus	26.3	26.3	77%	201	94.12%	6396	XM_006525768.5
PREDICTED: Mus musculus predicted gene, 31439 (Gm31439), transcript variant X2, ncRNA	Mus musculus	26.3	26.3	77%	201	94.12%	7356	XR_004939945.1
PREDICTED: Mus musculus zinc finger protein 40 (Zfp40), transcript variant X3, mRNA	Mus musculus	26.3	26.3	59%	201	100.00%	11684	XM_030249763.2
PREDICTED: Mus musculus N-acetylglucosamine-1-phosphotransferase, gamma subunit (Gnptg), transc...	Mus musculus	26.3	26.3	59%	201	100.00%	2050	XM_036163463.1
PREDICTED: Mus musculus N-acetylglucosamine-1-phosphotransferase, gamma subunit (Gnptg), transc...	Mus musculus	26.3	26.3	59%	201	100.00%	2047	XR_004939505.1
PREDICTED: Mus musculus N-acetylglucosamine-1-phosphotransferase, gamma subunit (Gnptg), transc...	Mus musculus	26.3	26.3	59%	201	100.00%	3381	XM_017317383.3
PREDICTED: Mus musculus interleukin 1 receptor accessory protein (Il1rap), transcript variant X1, mRNA	Mus musculus	26.3	26.3	59%	201	100.00%	9179	XM_036159791.1
PREDICTED: Mus musculus predicted gene, 41348 (Gm41348), ncRNA	Mus musculus	26.3	26.3	59%	201	100.00%	1749	XR_875429.3
PREDICTED: Mus musculus SECIS binding protein 2 (Secisbp2), transcript variant X6, mRNA	Mus musculus	26.3	26.3	59%	201	100.00%	3295	XM_006516966.3
PREDICTED: Mus musculus SECIS binding protein 2 (Secisbp2), transcript variant X5, misc_RNA	Mus musculus	26.3	26.3	59%	201	100.00%	2307	XR_003950489.2
PREDICTED: Mus musculus SECIS binding protein 2 (Secisbp2), transcript variant X4, misc_RNA	Mus musculus	26.3	26.3	59%	201	100.00%	3095	XR_003950488.2
PREDICTED: Mus musculus SECIS binding protein 2 (Secisbp2), transcript variant X3, misc_RNA	Mus musculus	26.3	26.3	59%	201	100.00%	2796	XR_382238.5
PREDICTED: Mus musculus SECIS binding protein 2 (Secisbp2), transcript variant X2, misc_RNA	Mus musculus	26.3	26.3	59%	201	100.00%	3343	XR_382237.5
PREDICTED: Mus musculus SECIS binding protein 2 (Secisbp2), transcript variant X1, misc_RNA	Mus musculus	26.3	26.3	59%	201	100.00%	3602	XR_004938074.1
PREDICTED: Mus musculus transcription activation suppressor family member 2 (Tasor2), transcript vari...	Mus musculus	26.3	26.3	59%	201	100.00%	7526	XM_006515463.3
PREDICTED: Mus musculus transcription activation suppressor family member 2 (Tasor2), transcript vari...	Mus musculus	26.3	26.3	59%	201	100.00%	8137	XM_006515461.5
PREDICTED: Mus musculus predicted gene, 51009 (Gm51009), ncRNA	Mus musculus	26.3	26.3	59%	201	100.00%	52869	XR_003949603.2
PREDICTED: Mus musculus XIAP associated factor 1 (Xaf1), transcript variant X5, mRNA	Mus musculus	26.3	26.3	59%	201	100.00%	2292	XM_036156775.1
PREDICTED: Mus musculus XIAP associated factor 1 (Xaf1), transcript variant X4, mRNA	Mus musculus	26.3	26.3	59%	201	100.00%	2304	XM_017314514.3

Genomic sequences

Mus musculus strain C57BL/6J chromosome 1 unlocalized genomic scaffold, GRCm39 MMCHR1_RAND...	Mus musculus	28.2	52.5	68%	51	100.00%	153618	NT_166282.1
[illegible]	[illegible]	[illegible]	[illegible]	[illegible]	[illegible]	[illegible]	[illegible]	[illegible]

shRNA: {si16-ldb3a165V}
Target Sequence: GCATGTACTCACAGGATGTTAT

Transcripts

PREDICTED: Mus musculus LIM domain binding 3 (Ldb3), transcript variant X4, mRNA	Mus musculus	36.2	36.2	81%	0.21	100.00%	1497	XM_036519019.5
PREDICTED: Mus musculus LIM domain binding 3 (Ldb3), transcript variant X3, mRNA	Mus musculus	36.2	36.2	81%	0.21	100.00%	4728	XM_036519018.5
PREDICTED: Mus musculus LIM domain binding 3 (Ldb3), transcript variant X2, mRNA	Mus musculus	36.2	36.2	81%	0.21	100.00%	4929	XM_036519017.5
Mus musculus LIM domain binding 3 (Ldb3), transcript variant 6, mRNA	Mus musculus	36.2	36.2	81%	0.21	100.00%	1506	NM_001039075.2
Mus musculus LIM domain binding 3 (Ldb3), transcript variant 5, mRNA	Mus musculus	36.2	36.2	81%	0.21	100.00%	4954	NM_001039074.2
Mus musculus LIM domain binding 3 (Ldb3), transcript variant 4, mRNA	Mus musculus	36.2	36.2	81%	0.21	100.00%	4737	NM_001039073.2
Mus musculus LIM domain binding 3 (Ldb3), transcript variant 3, mRNA	Mus musculus	36.2	36.2	81%	0.21	100.00%	4908	NM_001039072.2
Mus musculus strain C57BL/6J chromosome 14, GRCm39	Mus musculus	36.2	6841	100%	0.21	100.00%	125139656	NC_000080.7
Mus musculus strain C57BL/6J chromosome X, GRCm39	Mus musculus	34.2	7059	100%	0.82	100.00%	169476592	NC_000086.8
Mus musculus strain C57BL/6J chromosome 5, GRCm39	Mus musculus	32.2	8039	100%	3.3	100.00%	151758149	NC_000071.7
Mus musculus strain C57BL/6J chromosome 6, GRCm39	Mus musculus	32.2	7475	100%	3.3	100.00%	149588044	NC_000072.7
Mus musculus strain C57BL/6J chromosome 7, GRCm39	Mus musculus	32.2	6902	100%	3.3	100.00%	144995196	NC_000073.7
Mus musculus strain C57BL/6J chromosome 8, GRCm39	Mus musculus	32.2	6483	100%	3.3	100.00%	130127694	NC_000074.7
Mus musculus strain C57BL/6J chromosome 9, GRCm39	Mus musculus	32.2	6681	100%	3.3	100.00%	124359700	NC_000075.7
Mus musculus strain C57BL/6J chromosome 2, GRCm39	Mus musculus	32.2	11379	100%	3.3	100.00%	181755017	NC_000068.8
Mus musculus strain C57BL/6J chromosome 3, GRCm39	Mus musculus	32.2	8110	100%	3.3	100.00%	159745316	NC_000069.7
Mus musculus strain C57BL/6J chromosome 4, GRCm39	Mus musculus	32.2	7161	100%	3.3	100.00%	156860686	NC_000070.7
PREDICTED: Mus musculus thyroid hormone receptor interactor 11 (Trip11), transcript variant X4, mRNA	Mus musculus	30.2	30.2	68%	13	100.00%	11764	XM_036157159.1
PREDICTED: Mus musculus thyroid hormone receptor interactor 11 (Trip11), transcript variant X3, misc_...	Mus musculus	30.2	30.2	68%	13	100.00%	9708	XR_004937402.1
PREDICTED: Mus musculus thyroid hormone receptor interactor 11 (Trip11), transcript variant X2, mRNA	Mus musculus	30.2	30.2	68%	13	100.00%	9683	XM_011243986.3
PREDICTED: Mus musculus thyroid hormone receptor interactor 11 (Trip11), transcript variant X1, mRNA	Mus musculus	30.2	30.2	68%	13	100.00%	9760	XM_036515382.4
Mus musculus thyroid hormone receptor interactor 11 (Trip11), mRNA	Mus musculus	30.2	30.2	68%	13	100.00%	6448	NM_028446.1
Mus musculus strain C57BL/6J chromosome 1, GRCm39	Mus musculus	30.2	11019	100%	13	100.00%	195154279	NC_000067.7
Mus musculus strain C57BL/6J chromosome 10, GRCm39	Mus musculus	30.2	6216	100%	13	100.00%	130530862	NC_000076.7
Mus musculus strain C57BL/6J chromosome 11, GRCm39	Mus musculus	30.2	5972	100%	13	100.00%	121973369	NC_000077.7
Mus musculus strain C57BL/6J chromosome 15, GRCm39	Mus musculus	30.2	5326	100%	13	100.00%	104073951	NC_000081.7
Mus musculus strain C57BL/6J chromosome 19, GRCm39	Mus musculus	30.2	3367	100%	13	100.00%	61420004	NC_000085.7
Mus musculus strain C57BL/6J chromosome 12, GRCm39	Mus musculus	30.2	6115	100%	13	100.00%	120092757	NC_000078.7
Mus musculus strain C57BL/6J chromosome 13, GRCm39	Mus musculus	30.2	6348	100%	13	100.00%	120883175	NC_000079.7
Mus musculus strain C57BL/6J chromosome 16, GRCm39	Mus musculus	30.2	4532	100%	13	94.74%	98008968	NC_000082.7
Mus musculus strain C57BL/6J chromosome 17, GRCm39	Mus musculus	30.2	5248	100%	13	100.00%	95294699	NC_000083.7
PREDICTED: Mus musculus casein kinase 1, gamma 1 (Csnk1g1), transcript variant X13, misc_RNA	Mus musculus	28.2	28.2	63%	51	100.00%	10328	XR_003947839.2
PREDICTED: Mus musculus casein kinase 1, gamma 1 (Csnk1g1), transcript variant X11, mRNA	Mus musculus	28.2	28.2	63%	51	100.00%	7201	XM_011242709.3
PREDICTED: Mus musculus casein kinase 1, gamma 1 (Csnk1g1), transcript variant X10, mRNA	Mus musculus	28.2	28.2	63%	51	100.00%	7192	XM_030244227.2
PREDICTED: Mus musculus casein kinase 1, gamma 1 (Csnk1g1), transcript variant X9, mRNA	Mus musculus	28.2	28.2	63%	51	100.00%	7287	XM_011242708.3
PREDICTED: Mus musculus casein kinase 1, gamma 1 (Csnk1g1), transcript variant X8, mRNA	Mus musculus	28.2	28.2	63%	51	100.00%	7415	XM_030244225.2
PREDICTED: Mus musculus casein kinase 1, gamma 1 (Csnk1g1), transcript variant X7, mRNA	Mus musculus	28.2	28.2	63%	51	100.00%	7479	XM_036154824.1
PREDICTED: Mus musculus casein kinase 1, gamma 1 (Csnk1g1), transcript variant X6, mRNA	Mus musculus	28.2	28.2	63%	51	100.00%	7216	XM_006511008.4
PREDICTED: Mus musculus casein kinase 1, gamma 1 (Csnk1g1), transcript variant X12, mRNA	Mus musculus	28.2	28.2	63%	51	100.00%	7177	XM_011242710.2
PREDICTED: Mus musculus casein kinase 1, gamma 1 (Csnk1g1), transcript variant X5, mRNA	Mus musculus	28.2	28.2	63%	51	100.00%	7263	XM_011242707.2
PREDICTED: Mus musculus casein kinase 1, gamma 1 (Csnk1g1), transcript variant X4, mRNA	Mus musculus	28.2	28.2	63%	51	100.00%	7365	XM_011242706.2
PREDICTED: Mus musculus casein kinase 1, gamma 1 (Csnk1g1), transcript variant X3, mRNA	Mus musculus	28.2	28.2	63%	51	100.00%	7247	XM_011242705.2
PREDICTED: Mus musculus casein kinase 1, gamma 1 (Csnk1g1), transcript variant X2, mRNA	Mus musculus	28.2	28.2	63%	51	100.00%	7430	XM_011242704.2
PREDICTED: Mus musculus casein kinase 1, gamma 1 (Csnk1g1), transcript variant X1, mRNA	Mus musculus	28.2	28.2	63%	51	100.00%	7312	XM_011242703.2
PREDICTED: Mus musculus predicted gene, 32342 (Gm32342), transcript variant X3, ncRNA	Mus musculus	28.2	28.2	63%	51	100.00%	4590	XR_003952929.1
PREDICTED: Mus musculus predicted gene, 30837 (Gm30837), transcript variant X2, ncRNA	Mus musculus	28.2	28.2	63%	51	100.00%	457	XR_376965.2
PREDICTED: Mus musculus predicted gene, 30837 (Gm30837), transcript variant X1, ncRNA	Mus musculus	28.2	28.2	63%	51	100.00%	483	XR_376966.2
Mus musculus casein kinase 1, gamma 1 (Csnk1g1), mRNA	Mus musculus	28.2	28.2	63%	51	100.00%	7183	NM_173185.2
Mus musculus strain C57BL/6J chromosome 18, GRCm39	Mus musculus	28.2	4354	100%	51	100.00%	90720763	NC_000084.7
PREDICTED: Mus musculus predicted gene, 38468 (Gm38468), transcript variant X2, ncRNA	Mus musculus	28.2	28.2	63%	51	100.00%	1245	XR_375892.4
PREDICTED: Mus musculus predicted gene, 38468 (Gm38468), transcript variant X1, ncRNA	Mus musculus	28.2	28.2	63%	51	100.00%	1509	XR_004941619.1
PREDICTED: Mus musculus predicted gene, 30986 (Gm30986), transcript variant X2, ncRNA	Mus musculus	28.2	28.2	63%	51	100.00%	1573	XR_374993.4
PREDICTED: Mus musculus predicted gene, 30986 (Gm30986), transcript variant X1, ncRNA	Mus musculus	28.2	28.2	63%	51	100.00%	1793	XR_374992.3
PREDICTED: Mus musculus predicted gene 12462 (Gm12462), transcript variant X2, ncRNA	Mus musculus	28.2	28.2	81%	51	94.44%	2864	XR_003955388.1
PREDICTED: Mus musculus predicted gene 12462 (Gm12462), transcript variant X1, ncRNA	Mus musculus	28.2	28.2	81%	51	94.44%	3166	XR_003955387.1
Mus musculus ATPase, H+ transporting, lysosomal V1 subunit B2 (Atp6v1b2), mRNA	Mus musculus	28.2	28.2	63%	51	100.00%	2742	NM_007509.3
Mus musculus ras homolog family member J (Rhoj), mRNA	Mus musculus	28.2	28.2	63%	51	100.00%	2358	NM_023275.2
PREDICTED: Mus musculus X-linked Kx blood group related, X-linked (Xkrx), transcript variant X6, mRNA	Mus musculus	26.3	26.3	59%	201	100.00%	2962	XM_036161993.1
PREDICTED: Mus musculus X-linked Kx blood group related, X-linked (Xkrx), transcript variant X5, mRNA	Mus musculus	26.3	26.3	59%	201	100.00%	4560	XM_006528570.5
PREDICTED: Mus musculus X-linked Kx blood group related, X-linked (Xkrx), transcript variant X4, mRNA	Mus musculus	26.3	26.3	59%	201	100.00%	4101	XM_036161992.1
PREDICTED: Mus musculus X-linked Kx blood group related, X-linked (Xkrx), transcript variant X3, mRNA	Mus musculus	26.3	26.3	59%	201	100.00%	3227	XM_006528569.5
PREDICTED: Mus musculus X-linked Kx blood group related, X-linked (Xkrx), transcript variant X2, mRNA	Mus musculus	26.3	26.3	59%	201	100.00%	3253	XM_017318525.3
PREDICTED: Mus musculus X-linked Kx blood group related, X-linked (Xkrx), transcript variant X1, mRNA	Mus musculus	26.3	26.3	59%	201	100.00%	2975	XM_006528568.5
PREDICTED: Mus musculus predicted gene, 26614 (Gm26614), transcript variant X3, ncRNA	Mus musculus	26.3	26.3	59%	201	100.00%	18638	XR_004935702.1
PREDICTED: Mus musculus predicted gene, 26614 (Gm26614), transcript variant X2, ncRNA	Mus musculus	26.3	26.3	59%	201	100.00%	17622	XR_004935701.1
PREDICTED: Mus musculus predicted gene, 26614 (Gm26614), transcript variant X1, ncRNA	Mus musculus	26.3	26.3	59%	201	100.00%	19736	XR_004935700.1
PREDICTED: Mus musculus Rho guanine nucleotide exchange factor (GEF) 12 (Arhgef12), transcript va...	Mus musculus	26.3	26.3	59%	201	100.00%	18790	XM_011242598.4
PREDICTED: Mus musculus Rho guanine nucleotide exchange factor (GEF) 12 (Arhgef12), transcript va...	Mus musculus	26.3	26.3	59%	201	100.00%	18783	XM_030244573.2
PREDICTED: Mus musculus Rho guanine nucleotide exchange factor (GEF) 12 (Arhgef12), transcript va...	Mus musculus	26.3	26.3	59%	201	100.00%	18840	XM_030244572.2
PREDICTED: Mus musculus Rho guanine nucleotide exchange factor (GEF) 12 (Arhgef12), transcript va...	Mus musculus	26.3	26.3	59%	201	100.00%	18786	XM_017313567.3
PREDICTED: Mus musculus Rho guanine nucleotide exchange factor (GEF) 12 (Arhgef12), transcript va...	Mus musculus	26.3	26.3	59%	201	100.00%	18843	XM_030244571.2
PREDICTED: Mus musculus uncharacterized LOC118567615 (LOC118567615), ncRNA	Mus musculus	26.3	26.3	59%	201	100.00%	647	XR_004935080.1
PREDICTED: Mus musculus zinc finger protein 930 (Zfp930), transcript variant X3, mRNA	Mus musculus	26.3	26.3	59%	201	100.00%	3079	XM_030243456.2
PREDICTED: Mus musculus zinc finger protein 930 (Zfp930), transcript variant X2, mRNA	Mus musculus	26.3	26.3	59%	201	100.00%	3865	XM_030243455.2
PREDICTED: Mus musculus zinc finger protein 930 (Zfp930), transcript variant X1, mRNA	Mus musculus	26.3	26.3	59%	201	100.00%	3049	XM_030243454.2
PREDICTED: Mus musculus early B cell factor 3 (Ebf3), transcript variant X28, mRNA	Mus musculus	26.3	26.3	59%	201	100.00%	4673	XM_036152653.1
PREDICTED: Mus musculus early B cell factor 3 (Ebf3), transcript variant X26, mRNA	Mus musculus	26.3	26.3	59%	201	100.00%	5874	XM_036152651.1
PREDICTED: Mus musculus early B cell factor 3 (Ebf3), transcript variant X25, mRNA	Mus musculus	26.3	26.3	59%	201	100.00%	5901	XM_036152650.1
PREDICTED: Mus musculus zinc finger protein 638 (Zfp638), transcript variant X7, misc_RNA	Mus musculus	26.3	26.3	77%	201	94.12%	7505	XR_004942919.1
PREDICTED: Mus musculus mitochondrial transcription termination factor 1a (Mterf1a), transcript variant...	Mus musculus	26.3	26.3	77%	201	94.12%	3596	XM_017320999.3
PREDICTED: Mus musculus mitochondrial transcription termination factor 1a (Mterf1a), transcript variant...	Mus musculus	26.3	26.3	77%	201	94.12%	3628	XM_017321000.2
PREDICTED: Mus musculus predicted gene, 30019 (Gm30019), transcript variant X2, ncRNA	Mus musculus	26.3	26.3	59%	201	100.00%	1532	XR_003955285.2
PREDICTED: Mus musculus Rho GTPase activating protein 21 (Arhgap21), transcript variant X20, mRNA	Mus musculus	26.3	26.3	59%	201	100.00%	18104	XM_017319263.3
PREDICTED: Mus musculus Rho GTPase activating protein 21 (Arhgap21), transcript variant X19, misc_...	Mus musculus	26.3	26.3	59%	201	100.00%	22243	XR_004940733.1
PREDICTED: Mus musculus Rho GTPase activating protein 21 (Arhgap21), transcript variant X18, misc_...	Mus musculus	26.3	26.3	59%	201	100.00%	21126	XR_373946.5
PREDICTED: Mus musculus Rho GTPase activating protein 21 (Arhgap21), transcript variant X17, misc_...	Mus musculus	26.3	26.3	59%	201	100.00%	21129	XR_373945.5
PREDICTED: Mus musculus Rho GTPase activating protein 21 (Arhgap21), transcript variant X16, misc_...	Mus musculus	26.3	26.3	59%	201	100.00%	22017	XR_004940732.1
PREDICTED: Mus musculus Rho GTPase activating protein 21 (Arhgap21), transcript variant X15, mRNA	Mus musculus	26.3	26.3	59%	201	100.00%	21263	XM_017319262.3
PREDICTED: Mus musculus Rho GTPase activating protein 21 (Arhgap21), transcript variant X14, mRNA	Mus musculus	26.3	26.3	59%	201	100.00%	21296	XM_011238986.4
PREDICTED: Mus musculus Rho GTPase activating protein 21 (Arhgap21), transcript variant X11, mRNA	Mus musculus	26.3	26.3	59%	201	100.00%	20821	XM_030252086.2
PREDICTED: Mus musculus Rho GTPase activating protein 21 (Arhgap21), transcript variant X8, mRNA	Mus musculus	26.3	26.3	59%	201	100.00%	20824	XM_006497544.5
PREDICTED: Mus musculus Rho GTPase activating protein 21 (Arhgap21), transcript variant X4, mRNA	Mus musculus	26.3	26.3	59%	201	100.00%	20851	XM_006497542.5
PREDICTED: Mus musculus Rho GTPase activating protein 21 (Arhgap21), transcript variant X1, mRNA	Mus musculus	26.3	26.3	59%	201	100.00%	20854	XM_006497538.5
PREDICTED: Mus musculus N-acetylglucosamine-1-phosphotransferase, gamma subunit (Gnptg), trans...	Mus musculus	26.3	26.3	59%	201	100.00%	2050	XM_036163463.1
PREDICTED: Mus musculus N-acetylglucosamine-1-phosphotransferase, gamma subunit (Gnptg), trans...	Mus musculus	26.3	26.3	59%	201	100.00%	2047	XR_004939505.1
PREDICTED: Mus musculus N-acetylglucosamine-1-phosphotransferase, gamma subunit (Gnptg), trans...	Mus musculus	26.3	26.3	59%	201	100.00%	3381	XM_017317383.3
PREDICTED: Mus musculus interleukin 1 receptor accessory protein (Il1rap), transcript variant X1, mRNA	Mus musculus	26.3	26.3	59%	201	100.00%	9179	XM_036159791.1
PREDICTED: Mus musculus predicted gene, 41348 (Gm41348), ncRNA	Mus musculus	26.3	26.3	59%	201	100.00%	1749	XR_875429.3
PREDICTED: Mus musculus myosin X (Myo10), transcript variant X1, mRNA	Mus musculus	26.3	26.3	77%	201	94.12%	14651	XM_030248368.2
PREDICTED: Mus musculus predicted gene, 52128 (Gm52128), ncRNA	Mus musculus	26.3	26.3	77%	201	94.12%	36006	XR_003951168.2

shRNA: Scramble miR30-shRNA
Target Sequence: ACCTAAGGTTAAGTCGCCCTCG

Transcripts

Mus musculus strain C57BL/6J chromosome X, GRCm39	Mus musculus	32.2	1017	95%	3.3	100.00%	169476592	NC_000086.8
Mus musculus strain C57BL/6J chromosome 2, GRCm39	Mus musculus	30.2	1533	100%	13	100.00%	181755017	NC_000068.8
Mus musculus strain C57BL/6J chromosome 14, GRCm39	Mus musculus	30.2	1015	100%	13	100.00%	125139656	NC_000080.7
PREDICTED: Mus musculus predicted gene 13748 (Gm13748), ncRNA	Mus musculus	28.2	28.2	63%	51	100.00%	11052	XR_003947836.2
Mus musculus strain C57BL/6J chromosome 1, GRCm39	Mus musculus	28.2	1504	100%	51	100.00%	195154279	NC_000067.7
Mus musculus strain C57BL/6J chromosome 4, GRCm39	Mus musculus	28.2	1426	100%	51	100.00%	156860686	NC_000070.7
Mus musculus strain C57BL/6J chromosome 5, GRCm39	Mus musculus	28.2	1504	100%	51	100.00%	151758149	NC_000071.7
Mus musculus strain C57BL/6J chromosome 6, GRCm39	Mus musculus	28.2	1078	95%	51	100.00%	149588044	NC_000072.7
Mus musculus strain C57BL/6J chromosome 7, GRCm39	Mus musculus	28.2	1033	100%	51	100.00%	144995196	NC_000073.7
Mus musculus strain C57BL/6J chromosome 8, GRCm39	Mus musculus	28.2	1084	100%	51	100.00%	130127694	NC_000074.7
Mus musculus strain C57BL/6J chromosome 11, GRCm39	Mus musculus	28.2	1065	95%	51	100.00%	121973369	NC_000077.7
Mus musculus strain C57BL/6J chromosome 12, GRCm39	Mus musculus	28.2	1092	90%	51	100.00%	120092757	NC_000078.7
Mus musculus strain C57BL/6J chromosome 13, GRCm39	Mus musculus	28.2	1219	90%	51	94.44%	120883175	NC_000079.7
Mus musculus strain C57BL/6J chromosome 15, GRCm39	Mus musculus	28.2	693	95%	51	100.00%	104073951	NC_000081.7
Mus musculus strain C57BL/6J chromosome 16, GRCm39	Mus musculus	28.2	667	95%	51	100.00%	98008968	NC_000082.7
Mus musculus strain C57BL/6J chromosome 17, GRCm39	Mus musculus	28.2	780	95%	51	100.00%	95294699	NC_000083.7
Mus musculus strain C57BL/6J chromosome 19, GRCm39	Mus musculus	28.2	471	72%	51	100.00%	61420004	NC_000085.7
PREDICTED: Mus musculus mitoguardin 1 (Miga1), transcript variant X3, mRNA	Mus musculus	26.3	26.3	59%	201	100.00%	13109	XM_036163015.1
PREDICTED: Mus musculus ankyrin 2, brain (Ank2), transcript variant X43, mRNA	Mus musculus	26.3	26.3	59%	201	100.00%	32626	XM_036162848.1
PREDICTED: Mus musculus ankyrin 2, brain (Ank2), transcript variant X38, mRNA	Mus musculus	26.3	26.3	59%	201	100.00%	32656	XM_036162845.1
PREDICTED: Mus musculus ankyrin 2, brain (Ank2), transcript variant X35, mRNA	Mus musculus	26.3	26.3	59%	201	100.00%	32680	XM_036162842.1
PREDICTED: Mus musculus ankyrin 2, brain (Ank2), transcript variant X34, mRNA	Mus musculus	26.3	26.3	59%	201	100.00%	32680	XM_036162841.1
PREDICTED: Mus musculus ankyrin 2, brain (Ank2), transcript variant X31, mRNA	Mus musculus	26.3	26.3	59%	201	100.00%	32700	XM_036162838.1
PREDICTED: Mus musculus ankyrin 2, brain (Ank2), transcript variant X22, mRNA	Mus musculus	26.3	26.3	59%	201	100.00%	32755	XM_036162833.1
PREDICTED: Mus musculus ankyrin 2, brain (Ank2), transcript variant X19, mRNA	Mus musculus	26.3	26.3	59%	201	100.00%	32779	XM_036162830.1
PREDICTED: Mus musculus guanosine monophosphate reductase 2 (Gmpr2), transcript variant X2, mRNA	Mus musculus	26.3	26.3	59%	201	100.00%	6265	XM_011244882.4
PREDICTED: Mus musculus predicted gene, 52013 (Gm52013), transcript variant X2, ncRNA	Mus musculus	26.3	26.3	59%	201	100.00%	26661	XR_004937626.1
PREDICTED: Mus musculus predicted gene, 52013 (Gm52013), transcript variant X1, ncRNA	Mus musculus	26.3	26.3	59%	201	100.00%	21529	XR_004937625.1
PREDICTED: Mus musculus mitoguardin 1 (Miga1), transcript variant X4, misc_RNA	Mus musculus	26.3	26.3	59%	201	100.00%	5018	XR_001783676.2
PREDICTED: Mus musculus predicted gene, 46219 (Gm46219), ncRNA	Mus musculus	26.3	26.3	59%	201	100.00%	1695	XR_001779752.2
Mus musculus POU domain, class 4, transcription factor 3 (Pou4f3), mRNA	Mus musculus	26.3	26.3	59%	201	100.00%	1146	NM_138945.2
Mus musculus mitoguardin 1 (Miga1), transcript variant 2, mRNA	Mus musculus	26.3	26.3	59%	201	100.00%	5163	NM_001162375.1
Mus musculus mitoguardin 1 (Miga1), transcript variant 1, mRNA	Mus musculus	26.3	26.3	59%	201	100.00%	5355	NM_174868.4
Mus musculus strain C57BL/6J chromosome 3, GRCm39	Mus musculus	26.3	1286	95%	201	100.00%	159745316	NC_000069.7
Mus musculus strain C57BL/6J chromosome 9, GRCm39	Mus musculus	26.3	1031	100%	201	100.00%	124359700	NC_000075.7
Mus musculus strain C57BL/6J chromosome 10, GRCm39	Mus musculus	26.3	1349	100%	201	100.00%	130530862	NC_000076.7
Mus musculus strain C57BL/6J chromosome 18, GRCm39	Mus musculus	26.3	616	100%	201	100.00%	90720763	NC_000084.7
PREDICTED: Mus musculus RIKEN cDNA 4931406C07 gene (4931406C07Rik), transcript variant X3, m...	Mus musculus	24.3	24.3	54%	794	100.00%	2586	XM_030244585.2
PREDICTED: Mus musculus RIKEN cDNA 4931406C07 gene (4931406C07Rik), transcript variant X2, m...	Mus musculus	24.3	24.3	54%	794	100.00%	2749	XM_036155283.1
PREDICTED: Mus musculus RIKEN cDNA 4931406C07 gene (4931406C07Rik), transcript variant X1, m...	Mus musculus	24.3	24.3	54%	794	100.00%	2637	XM_006510596.3
PREDICTED: Mus musculus enhancer of mRNA decapping 3 (Edc3), transcript variant X3, mRNA	Mus musculus	24.3	24.3	54%	794	100.00%	5644	XM_011242760.4
PREDICTED: Mus musculus enhancer of mRNA decapping 3 (Edc3), transcript variant X1, mRNA	Mus musculus	24.3	24.3	54%	794	100.00%	4352	XM_036155089.1
PREDICTED: Mus musculus predicted gene, 32643 (Gm32643), transcript variant X15, ncRNA	Mus musculus	24.3	24.3	54%	794	100.00%	14103	XR_003947629.2
PREDICTED: Mus musculus predicted gene, 32643 (Gm32643), transcript variant X14, ncRNA	Mus musculus	24.3	24.3	54%	794	100.00%	14062	XR_003947628.2
PREDICTED: Mus musculus predicted gene, 32643 (Gm32643), transcript variant X13, ncRNA	Mus musculus	24.3	24.3	54%	794	100.00%	14157	XR_003947627.2
PREDICTED: Mus musculus predicted gene, 32643 (Gm32643), transcript variant X12, ncRNA	Mus musculus	24.3	24.3	54%	794	100.00%	13981	XR_003947626.2
PREDICTED: Mus musculus predicted gene, 32643 (Gm32643), transcript variant X11, ncRNA	Mus musculus	24.3	24.3	54%	794	100.00%	14008	XR_003947625.2
PREDICTED: Mus musculus predicted gene, 32643 (Gm32643), transcript variant X10, ncRNA	Mus musculus	24.3	24.3	54%	794	100.00%	13934	XR_879390.4
PREDICTED: Mus musculus predicted gene, 32643 (Gm32643), transcript variant X9, ncRNA	Mus musculus	24.3	24.3	54%	794	100.00%	14401	XR_004935174.1
PREDICTED: Mus musculus predicted gene, 32643 (Gm32643), transcript variant X8, ncRNA	Mus musculus	24.3	24.3	54%	794	100.00%	14251	XR_004935173.1
PREDICTED: Mus musculus predicted gene, 32643 (Gm32643), transcript variant X7, ncRNA	Mus musculus	24.3	24.3	54%	794	100.00%	14056	XR_879387.4
PREDICTED: Mus musculus predicted gene, 32643 (Gm32643), transcript variant X6, ncRNA	Mus musculus	24.3	24.3	54%	794	100.00%	14103	XR_388237.5
PREDICTED: Mus musculus predicted gene, 32643 (Gm32643), transcript variant X5, ncRNA	Mus musculus	24.3	24.3	54%	794	100.00%	13953	XR_879386.4
PREDICTED: Mus musculus predicted gene, 32643 (Gm32643), transcript variant X4, ncRNA	Mus musculus	24.3	24.3	54%	794	100.00%	14419	XR_004935172.1
PREDICTED: Mus musculus predicted gene, 32643 (Gm32643), transcript variant X3, ncRNA	Mus musculus	24.3	24.3	54%	794	100.00%	14269	XR_004935171.1
PREDICTED: Mus musculus predicted gene, 32643 (Gm32643), transcript variant X2, ncRNA	Mus musculus	24.3	24.3	54%	794	100.00%	13906	XR_879384.4
PREDICTED: Mus musculus predicted gene, 32643 (Gm32643), transcript variant X1, ncRNA	Mus musculus	24.3	24.3	54%	794	100.00%	13886	XR_879385.4
PREDICTED: Mus musculus complement component 5a receptor 2 (C5ar2), transcript variant X9, mRNA	Mus musculus	24.3	24.3	54%	794	100.00%	2185	XM_006540056.3
PREDICTED: Mus musculus complement component 5a receptor 2 (C5ar2), transcript variant X8, mRNA	Mus musculus	24.3	24.3	54%	794	100.00%	2542	XM_006540055.5
PREDICTED: Mus musculus complement component 5a receptor 2 (C5ar2), transcript variant X7, mRNA	Mus musculus	24.3	24.3	54%	794	100.00%	2514	XM_006540054.5
PREDICTED: Mus musculus complement component 5a receptor 2 (C5ar2), transcript variant X1, misc_...	Mus musculus	24.3	24.3	54%	794	100.00%	2192	XR_881730.4
PREDICTED: Mus musculus potassium voltage-gated channel, Shal-related family, member 2 (Kcnd2), tr...	Mus musculus	24.3	24.3	54%	794	100.00%	95035	XM_036165884.1
PREDICTED: Mus musculus predicted gene, 52764 (Gm52764), transcript variant X1, ncRNA	Mus musculus	24.3	24.3	54%	794	100.00%	7721	XR_004942721.1
PREDICTED: Mus musculus predicted gene, 19417 (Gm19417), ncRNA	Mus musculus	24.3	24.3	54%	794	100.00%	26255	XR_376437.5
PREDICTED: Mus musculus predicted gene 11802 (Gm11802), transcript variant X7, ncRNA	Mus musculus	24.3	24.3	54%	794	100.00%	10637	XR_881093.4
PREDICTED: Mus musculus predicted gene 11802 (Gm11802), transcript variant X6, ncRNA	Mus musculus	24.3	24.3	54%	794	100.00%	10733	XR_881092.4
PREDICTED: Mus musculus predicted gene 11802 (Gm11802), transcript variant X3, ncRNA	Mus musculus	24.3	24.3	54%	794	100.00%	10797	XR_881089.4
PREDICTED: Mus musculus predicted gene 11802 (Gm11802), transcript variant X2, ncRNA	Mus musculus	24.3	24.3	54%	794	100.00%	10785	XR_881088.4
PREDICTED: Mus musculus predicted gene 11802 (Gm11802), transcript variant X1, ncRNA	Mus musculus	24.3	24.3	54%	794	100.00%	10661	XR_881090.4
PREDICTED: Mus musculus alkB homolog 3, alpha-ketoglutarate-dependent dioxygenase (Alkbh3), trans...	Mus musculus	24.3	24.3	54%	794	100.00%	11519	XM_006500125.4
PREDICTED: Mus musculus predicted gene, 30620 (Gm30620), transcript variant X6, ncRNA	Mus musculus	24.3	24.3	54%	794	100.00%	3760	XR_004939998.1
PREDICTED: Mus musculus predicted gene, 30620 (Gm30620), transcript variant X4, ncRNA	Mus musculus	24.3	24.3	54%	794	100.00%	4531	XR_004939997.1
PREDICTED: Mus musculus predicted gene, 30620 (Gm30620), transcript variant X1, ncRNA	Mus musculus	24.3	24.3	54%	794	100.00%	4534	XR_004939996.1
PREDICTED: Mus musculus protocadherin 9 (Pcdh9), transcript variant X2, misc_RNA	Mus musculus	24.3	24.3	54%	794	100.00%	148183	XR_003950833.2
PREDICTED: Mus musculus prospero homeobox 2 (Prox2), transcript variant X4, mRNA	Mus musculus	24.3	24.3	54%	794	100.00%	3910	XM_017315213.3
PREDICTED: Mus musculus prospero homeobox 2 (Prox2), transcript variant X2, mRNA	Mus musculus	24.3	24.3	54%	794	100.00%	8282	XM_030246952.2
PREDICTED: Mus musculus prospero homeobox 2 (Prox2), transcript variant X1, mRNA	Mus musculus	24.3	24.3	54%	794	100.00%	4350	XM_006516278.5
PREDICTED: Mus musculus myosin phosphatase Rho interacting protein (Mprip), transcript variant X2, m...	Mus musculus	24.3	24.3	54%	794	100.00%	16540	XR_003949455.2
PREDICTED: Mus musculus vascular endothelial zinc finger 1 (Vezf1), transcript variant X9, mRNA	Mus musculus	24.3	24.3	54%	794	100.00%	7355	XM_030245929.2
PREDICTED: Mus musculus vascular endothelial zinc finger 1 (Vezf1), transcript variant X8, mRNA	Mus musculus	24.3	24.3	54%	794	100.00%	6041	XM_030245928.2
PREDICTED: Mus musculus vascular endothelial zinc finger 1 (Vezf1), transcript variant X7, mRNA	Mus musculus	24.3	24.3	54%	794	100.00%	7158	XM_030245927.2
PREDICTED: Mus musculus vascular endothelial zinc finger 1 (Vezf1), transcript variant X6, mRNA	Mus musculus	24.3	24.3	54%	794	100.00%	7190	XM_011248969.3
PREDICTED: Mus musculus vascular endothelial zinc finger 1 (Vezf1), transcript variant X5, mRNA	Mus musculus	24.3	24.3	54%	794	100.00%	7708	XM_036156629.1
PREDICTED: Mus musculus vascular endothelial zinc finger 1 (Vezf1), transcript variant X4, mRNA	Mus musculus	24.3	24.3	54%	794	100.00%	7726	XM_036156628.1
PREDICTED: Mus musculus vascular endothelial zinc finger 1 (Vezf1), transcript variant X3, mRNA	Mus musculus	24.3	24.3	54%	794	100.00%	4951	XM_011248968.4
PREDICTED: Mus musculus vascular endothelial zinc finger 1 (Vezf1), transcript variant X2, mRNA	Mus musculus	24.3	24.3	54%	794	100.00%	5644	XM_036156627.1
PREDICTED: Mus musculus vascular endothelial zinc finger 1 (Vezf1), transcript variant X1, mRNA	Mus musculus	24.3	24.3	54%	794	100.00%	5662	XM_036156626.1
PREDICTED: Mus musculus predicted gene, 51801 (Gm51801), transcript variant X2, ncRNA	Mus musculus	24.3	24.3	54%	794	100.00%	7561	XR_003949003.2
PREDICTED: Mus musculus predicted gene, 51801 (Gm51801), transcript variant X1, ncRNA	Mus musculus	24.3	24.3	54%	794	100.00%	6458	XR_004936324.1
PREDICTED: Mus musculus NUAK family, SNF1-like kinase, 1 (Nuak1), transcript variant X2, mRNA	Mus musculus	24.3	24.3	54%	794	100.00%	5206	XM_011243603.4
PREDICTED: Mus musculus NUAK family, SNF1-like kinase, 1 (Nuak1), transcript variant X1, mRNA	Mus musculus	24.3	24.3	54%	794	100.00%	11216	XM_036156104.1
PREDICTED: Mus musculus TBC1 domain family, member 30 (Tbc1d30), transcript variant X1, mRNA	Mus musculus	24.3	24.3	54%	794	100.00%	5447	XM_030245334.2
PREDICTED: Mus musculus LEM domain containing 3 (Lemd3), transcript variant X1, misc_RNA	Mus musculus	24.3	24.3	54%	794	100.00%	4917	XR_001779544.3
PREDICTED: Mus musculus TAFA chemokine like family member 2 (Tafa2), transcript variant X5, mRNA	Mus musculus	24.3	24.3	54%	794	100.00%	18141	XM_036155794.1
PREDICTED: Mus musculus TAFA chemokine like family member 2 (Tafa2), transcript variant X4, mRNA	Mus musculus	24.3	24.3	54%	794	100.00%	17894	XM_030245097.2
PREDICTED: Mus musculus TAFA chemokine like family member 2 (Tafa2), transcript variant X3, mRNA	Mus musculus	24.3	24.3	54%	794	100.00%	18249	XM_036155793.1

Selected shRNA and Target Sequences			
**shRNA**	**Target Sequence in Mouse**	**Potential Off-Target mRNA (Mice)**	**Sequence Homology (%)**
		Mitoguardin 1 (Miga1)	59
shSCRNAmiR	ACCTAAGGTTAAGTCGCCCTCG	Ankyrin 2, brain (Ank2)	59
		POU domain, class 4, transcription factor 3 (Pou4f3)	59
		Coiled-coil domain containing 97 (Ccdc97)	81
sh10RNAmiR	ACTCACAGGATGTTATCATGGA	Thyroid hormone receptor interactor 11 (Trip11)	63
		ATPase, H+ transporting, lysosomal V1 subunit B2 (Atp6v1b2)	63
		Thyroid hormone receptor interactor 11 (Trip11)	68
sh16RNAmiR	GCATGTACTCACAGGATGTTAT	Casein Kinase 1, gamma 1 (Csnk1g1)	63
		ATPase, H+ transporting, lysosomal V1 subunit B2 (Atp6v1b2)	63

Therapeutic shRNAs were designed in a miR30 backbone for allele-specific silencing of the mutant *Ldb3* transcript, with in silico screening revealing only partial homology (≤68%) to unrelated mouse transcripts and no high-risk off-target matches.

A scramble shRNA with comparable homology did not produce molecular or functional effects, and two independent shRNAs yielded concordant outcomes, supporting target-specific silencing.

pAAV[miR30]-CMV>EGFP:{si10-Ldb3A165V}:WPRE

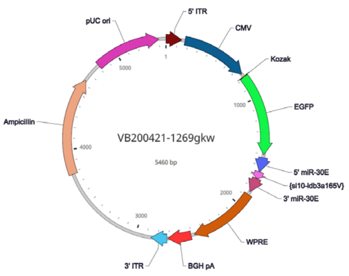

pAAV[miR30]-CMV>EGFP:{si16-ldb3a165V}:WPRE

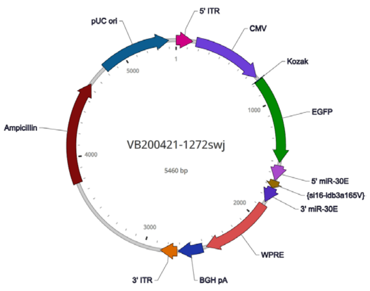

pAAV[miR30]-CMV>EGFP:Scramble_miR30-shRNA:WPRE

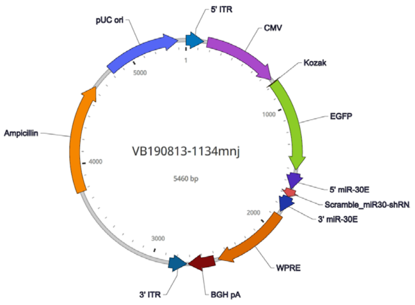

S. No.	Protein	Modifications (all sites)
1	SLC25A4	Y191, Y195, T197
2	ACTN2	S840
3	ANKRD2	S36
4	CASQ1	Y57
5	CA3	S48, Y51
6	CAVIN1	S38, T40, S42
7	CKM	S164, T166, S372, T322, T327, S337
8	SPAG9	T365
9	ALDH4A1	T541, S542
10	DES	S437
11	EEF1D	S162
12	FLNC	S2234, S2237
13	ALDOA	S36, T37, S39
14	PYGM	S514
15	GYS1	S652, S653, S657
16	HSP90AB1	S226, S255
17	HSPA12B	S276
18	JSRP1	S223, S225, S228, S232
19	JPH1	T460, T461, S469, S473, S475, S480
20	LDB3	S98, S179
21	ACSL1	S423, S424
22	LDHA	T322
23	MARCKS	S138, S141, T143
24	FXYD1	S82, S83
25	PHKB	S692, S693
26	ATP2A1	S547
27	SPEG	S542, S545, T546, S2322, S2323, S2325, S2327, S2333
28	SYNPO2L	T702
29	SYNPO2	S895, S899
30	TTN	S264, T266, S269, T299, S301
31	TPM1	S174
32	UBAC1	S98
33	VDAC1	S115, S117
34	VDAC2	T114, S116
35	CACNB1	T418

CYTO_pho	CYTO_tota	Annotated	Modificati	Confidenc	# Proteins	# PSMs	Master Pro	Positions i	Master Pro	Modificati	# Missed C	m/z [Da] (I	Theo. MH+	DeltaM [pp	XCorr (by S	Log2_Mea	Log2_Mea	Linear_Dif	Welch_Tte	**Welch_Ttest_Corrected_Pvalue**
CYTO_pho	CYTO_tota	[K].DIKHD	1xPhosph	High	1	9	P16015	P16015 [3	Carbonic a	P16015 1x	1	823.678	3291.69	0.54	6.53	0.00388	0.00888	2.28671	2.94E-05	0.00642
CYTO_pho	CYTO_tota	[K].LEKGQ	1xPhosph	High	1	8	P07310	P07310 [3	Creatine k	P07310 1x	1	894.485	2681.44	0.3	4.34	0.00085	0.00138	1.63628	3.46E-05	0.00642
CYTO_pho	CYTO_tota	[K].AEDGA	1xPhosph	High	1	10	P26645	P26645 [1	Myristoyla	P26645 1x	0	711.351	2132.04	-0.45	5.43	0.3704	0.7839	2.11639	3.90E-05	0.00642
CYTO_pho	CYTO_tota	[K].ATHPP	1xPhosph	High	1	5	Q8R3Z5	Q8R3Z5 [4	Voltage-de	Q8R3Z5 1	0	695.027	2083.07	-0.13	4.37	0.03769	0.0259	-1.45534	4.34E-05	0.00642
CYTO_pho	CYTO_tota	[K].ASSEG	1xPhosph	High	1	6	Q9JKS4	Q9JKS4 [1	LIM domai	Q9JKS4 1x	0	631.659	1892.96	0.28	5.28	0.01157	0.03207	2.77109	0.00014	0.01007
CYTO_pho	CYTO_tota	[R].HSSPH	1xPhosph	High	1	9	Q9Z1E4	Q9Z1E4 [6	Glycogen	Q9Z1E4 1	0	683.294	2047.87	-0.55	4.27	0.03094	0.01141	-2.71246	0.00014	0.01007
CYTO_pho	CYTO_tota	[K].LSVEA	1xPhosph	High	1	19	P07310	P07310 [1	Creatine k	P07310 1x	1	672.13	2685.5	-2.19	5.26	0.0025	0.00671	2.6834	0.00014	0.01007
CYTO_pho	CYTO_tota	[K].GILAAD	1xPhosph	High	1	59	P05064	P05064 [2	Fructose-b	P05064 1x	1	545.051	2177.18	-0.2	4.5	0.02979	0.04531	1.52096	0.00014	0.01007
CYTO_pho	CYTO_tota	[R].TGEPD	1xPhosph	High	1	12	Q9Z239	Q9Z239 [7	Phosphole	Q9Z239 1	1	732.004	2194	-0.31	5.42	2.14629	5.3525	2.49384	0.00015	0.01007
CYTO_pho	CYTO_tota	[K].RVPSP	1xPhosph	High	1	4	Q8VDI7	Q8VDI7 [9	Ubiquitin-	Q8VDI7 1x	1	527.984	1581.94	-0.46	3.16	0.33048	0.45704	1.38294	0.00017	0.01011
CYTO_pho	CYTO_tota	[K].IMSVIK	1xPhosph	High	1	2	Q8R429	Q8R429 [5	Sarcoplas	Q8R429 1	1	694.375	2081.11	0.4	3.22	0.00058	0.00136	2.32018	0.00019	0.01035
CYTO_pho	CYTO_tota	[R].AQSPT	2xPhosph	High	1	5	Q91YE8	Q91YE8 [8	Synaptopo	Q91YE8 2	0	713.357	2138.06	-0.85	3.49	1.03749	1.61731	1.55887	0.00034	0.01622
CYTO_pho	CYTO_tota	[R].SAPGK	1xPhosph	High	1	2	Q9WV06	Q9WV06 [	Ankyrin re	Q9WV06 1	1	866.442	2597.31	0.59	1.54	0.02331	0.00971	-2.3996	0.00037	0.01622
CYTO_pho	CYTO_tota	[R].EPGEP	2xPhosph	High	1	2	Q62407	Q62407 [5	Striated m	Q62407 2	0	602.554	2407.2	-0.36	2.41	0.1145	0.09583	-1.19486	0.00042	0.01622
CYTO_pho	CYTO_tota	[K].LTFDS	1xPhosph	High	1	9	Q60932	Q60932 [1	Voltage-de	Q60932 1	0	1045.03	2089.05	1.34	4.36	0.02691	0.01613	-1.66874	0.00043	0.01622
CYTO_pho	CYTO_tota	[K].QSHSF	1xPhosph	High	1	8	Q9ET80	Q9ET80 [4	Junctophil	Q9ET80 1x	0	727.7	2181.08	0.36	5.39	0.07118	0.06055	-1.17554	0.00044	0.01622
CYTO_pho	CYTO_tota	[R].EKEISD	1xPhosph	High	1	4	P11499	P11499 [2	Heat shoc	P11499 1x	1	848.424	2543.26	-0.13	3.35	0.00718	0.00526	-1.36383	0.00062	0.02121
CYTO_pho	CYTO_tota	[K].NYKNV	1xPhosph	High	1	3	O09165	O09165 [5	Calseques	O09165 1	1	800.941	1600.87	0.05	1.6	0.0023	0.00317	1.37887	0.00066	0.02121
CYTO_pho	CYTO_tota	[K].GATPA	1xPhosph	High	1	8	P57776	P57776 [1	Elongation	P57776 1x	2	838.596	4188.94	1.38	6.2	0.15271	0.11503	-1.32756	0.00068	0.02121
CYTO_pho	CYTO_tota	[K].GSSTP	1xPhosph	High	1	2	Q58A65	Q58A65 [3	C-Jun-ami	Q58A65 1	1	737.745	2211.22	-0.22	2.85	0.41429	0.72968	1.76127	0.00076	0.0226
CYTO_pho	CYTO_tota	[R].SAPGK	1xPhosph	High	1	2	Q9WV06	Q9WV06 [	Ankyrin re	Q9WV06 1	2	689.109	2753.41	0.59	2.31	0.02331	0.01196	-1.94981	0.00097	0.0274
CYTO_pho	CYTO_tota	[R].IGEDY	1xPhosph	High	1	4	Q9WUB3	Q9WUB3	Glycogen	Q9WUB3	1	785.081	2353.23	-0.49	3.8	0.00083	0.00119	1.4326	0.00106	0.02861
CYTO_pho	CYTO_tota	[R].GGSIS	1xPhosph	High	1	46	Q3MI48	Q3MI48 [2	Junctional	Q3MI48 1x	0	711.001	2130.99	-0.02	6.09	0.51815	0.39041	-1.3272	0.00133	0.03412
CYTO_pho	CYTO_tota	[R].SIDSS	1xPhosph	High	1	2	Q9CZJ2	Q9CZJ2 [2	Heat shoc	Q9CZJ2 1x	0	598.288	1195.57	0.7	1.85	0.28375	0.23102	-1.22826	0.0015	0.03631
CYTO_pho	CYTO_tota	[R].WTSPC	1xPhosph	High	1	14	Q8CHT0	Q8CHT0 [	Delta-1-py	Q8CHT0 1	0	549.644	1646.92	0.78	2.61	0.32183	0.21367	-1.50621	0.00153	0.03631
CYTO_pho	CYTO_tota	[K].SADTL	1xPhosph	High	1	6	P06151	P06151 [3	L-lactate d	P06151 1x	0	903.984	1806.96	-2.38	2.99	0.00177	0.00207	1.16848	0.00176	0.04016
CYTO_pho	CYTO_tota	[R].ATEEP	1xPhosph	High	1	21	O54724	O54724 [3	Caveolae-	O54724 1	0	741.374	2222.11	-0.09	5.2	0.12476	0.08362	-1.49197	0.0021	0.04279
CYTO_pho	CYTO_tota	[K].IQSSL	1xPhosph	High	1	4	P41216	P41216 [4	Long-chai	P41216 1x	0	739.417	1477.83	-0.42	1.57	0.00619	0.00509	-1.21764	0.00211	0.04279
CYTO_pho	CYTO_tota	[K].LTFDT	1xPhosph	High	1	4	Q60930	Q60930 [1	Voltage-de	Q60930 1	1	638.102	2549.38	0.67	3.82	0.01124	0.01438	1.27928	0.00215	0.04279
CYTO_pho	CYTO_tota	[R].AAYFG	1xPhosph	High	1	15	P48962	P48962 [1	ADP/ATP t	P48962 1x	0	947.486	1893.96	0.25	3.73	0.01424	0.01145	-1.24316	0.00217	0.04279
CYTO_pho	CYTO_tota	[K].GTTPP	2xPhosph	High	1	2	Q9ET80	Q9ET80 [4	Junctophil	Q9ET80 2x	1	528.013	2109.03	0.54	2.32	0.076	0.05987	-1.26942	0.00275	0.04915
CYTO_pho	CYTO_tota	[K].GILAAD	1xPhosph	High	1	191	P05064	P05064 [2	Fructose-b	P05064 1x	0	674.365	2021.08	0	5.13	0.04583	0.03983	-1.15067	0.00275	0.04915
CYTO_pho	CYTO_tota	[K].SKRPIF	1xPhosph	High	1	3	Q9JKS4	Q9JKS4 [8	LIM domai	Q9JKS4 1x	1	678.996	3390.95	-0.09	4.59	0.01165	0.02208	1.8954	0.00289	0.04915
CYTO_pho	CYTO_tota	[K].IEDVG	1xPhosph	High	1	20	P11499	P11499 [2	Heat shoc	P11499 1x	0	1092	2182.99	-0.39	4.2	0.03791	0.02568	-1.4762	0.0029	0.04915
CYTO_pho	CYTO_tota	[R].QSSTA	1xPhosph	High	1	6	Q7TSH2	Q7TSH2 [6	Phosphory	Q7TSH2 1	0	1028.84	3084.52	-0.01	5.84	0.02055	0.02738	1.33244	0.00291	0.04915
CYTO_pho	CYTO_tota	[R].GTGG	1xPhosph	High	1	11	P07310	P07310 [3	Creatine k	P07310 1x	0	793.051	2377.14	0.71	7.11	0.00051	0.00045	-1.13997	0.00368	0.05881
CYTO_pho	CYTO_tota	[R].LGSFG	1xPhosph	High	1	11	Q8VHX6	Q8VHX6 [	Filamin-C	Q8VHX6 1	0	661.346	1321.68	0.32	2.76	0.02556	0.03509	1.37309	0.05975	0.18141

CYTO_phospho_index	CYTO_total_index	Annotated Sequence	Modifications	[illegible]
CYTO_phospho_362	CYTO_total_12	[illegible]	[illegible]	[illegible]
[illegible]	[illegible]	[illegible]	[illegible]	[illegible]
CYTO_phospho_448	CYTO_total_3053	[illegible]	[illegible]	[illegible]
[illegible]	[illegible]	[illegible]	[illegible]	[illegible]
CYTO_phospho_1523	CYTO_total_360	[illegible]	[illegible]	[illegible]
[illegible]	[illegible]	[illegible]	[illegible]	[illegible]
CYTO_phospho_270	CYTO_total_1	[illegible]	[illegible]	[illegible]
[illegible]	[illegible]	[illegible]	[illegible]	[illegible]
[illegible]	[illegible]	[illegible]	[illegible]	[illegible]
[illegible]	[illegible]	[illegible]	[illegible]	[illegible]
[illegible]	[illegible]	[illegible]	[illegible]	[illegible]
[illegible]	[illegible]	[illegible]	[illegible]	[illegible]
[illegible]	[illegible]	[illegible]	[illegible]	[illegible]
CYTO_phospho_855	CYTO_total_44	[illegible]	[illegible]	[illegible]
[illegible]	[illegible]	[illegible]	[illegible]	[illegible]
CYTO_phospho_110	CYTO_total_28	[illegible]	[illegible]	[illegible]
[illegible]	[illegible]	[illegible]	[illegible]	[illegible]
CYTO_phospho_1502	CYTO_total_146	[illegible]	[illegible]	[illegible]
[illegible]	[illegible]	[illegible]	[illegible]	[illegible]
CYTO_phospho_1140	CYTO_total_182	[illegible]	[illegible]	[illegible]
[illegible]	[illegible]	[illegible]	[illegible]	[illegible]
CYTO_phospho_1524	CYTO_total_360	[illegible]	[illegible]	[illegible]
[illegible]	[illegible]	[illegible]	[illegible]	[illegible]
[illegible]	[illegible]	[illegible]	[illegible]	[illegible]
[illegible]	[illegible]	[illegible]	[illegible]	[illegible]
[illegible]	[illegible]	[illegible]	[illegible]	[illegible]
CYTO_phospho_923	CYTO_total_823	[illegible]	[illegible]	[illegible]
[illegible]	[illegible]	[illegible]	[illegible]	[illegible]
CYTO_phospho_33	CYTO_total_252	[illegible]	[illegible]	[illegible]
[illegible]	[illegible]	[illegible]	[illegible]	[illegible]
[illegible]	[illegible]	[illegible]	[illegible]	[illegible]

MWD_phospho_index	MWD_total_index	Annotated Sequence	Modifications	Confidence (by Search Engine): Sequest HT	# Proteins	# PSMs	Master Protein Accessions	Positions in Master Proteins	Master Protein Descriptions	Modifications in Master Proteins (all Sites)	# Missed Cleavages	m/z [Da] (by Search Engine): Sequest HT	Theo. MH+ [Da]	DeltaM [ppm] (by Search Engine): Sequest HT	XCorr (by Search Engine): Sequest HT	Log2_Mean_M_SC	Log2_Mean_M_TR	Linear Difference M_TR vs. M_SC	Welch_Ttest_Uncorrected_Pvalue	Welch_Ttest_Corrected_Pvalue
MWD_phospho_1536	MWD_total_25	[K].SKRPIPISTTAPPIQSPLPVIPHQK.[D]	1xPhospho [S16]; 2xTMTpro [K2; K25]; 1xTMTpro [N-Term]	High	1	8	Q9JKS4	Q9JKS4 [83-107]	LIM domain-binding protein 3 OS=Mus musculus OX=10090 GN=Ldb3 PE=1 SV=1	Q9JKS4 1xPhospho [S98]	1	739.83263	3695.15935	-6.84	7.99	0.008798067	0.013269286	1.508204778	1.19E-05	0.010119554
MWD_phospho_1351	MWD_total_71	[K].TLPQVSPKTPRPMAPK.[T]	1xPhospho [T9]; 1xOxidation [M13]; 2xTMTpro [K8; K16]; 1xTMTpro [N-Term]	High	1	6	Q8BWB1	Q8BWB1 [694-709]	Synaptopodin 2-like protein OS=Mus musculus OX=10090 GN=Synpo2l PE=1 SV=1	Q8BWB1 1xPhospho [T702]	1	675.13811	2697.52207	-0.17	5.96	0.016465902	0.028326162	1.7202922	4.88E-05	0.020772792
MWD_phospho_813	MWD_total_20	[R].ETSPEQRGSEVHTK.[K]	1xPhospho [S9]; 1xTMTpro [K14]; 1xTMTpro [N-Term]	High	1	3	P31001	P31001 [429-442]	Desmin OS=Mus musculus OX=10090 GN=Des PE=1 SV=3	P31001 1xPhospho [S437]	1	569.0408	2273.14186	-0.21	3.54	0.00284765	0.002199486	-1.294699164	8.20E-05	0.023260284
MWD_phospho_1179	MWD_total_91	[R].VPTVPPRPGSSLSGSIENLESEAVFEAK.[E]	3xPhospho [S13; S]; 1xTMTpro [K28]; 1xTMTpro [N-Term]	High	1	10	Q62407	Q62407 [2313-2340]	Striated muscle-specific serine/threonine-protein kinase OS=Mus musculus OX=10090 GN=Speg PE=1 SV=2	Q62407 3xPhospho [S2322; S2323; S2325; S2327; S2333]	0	937.20624	3745.80238	0.2	4.91	0.025542299	0.030877748	1.208886804	0.000169671	0.036097426
MWD_phospho_1485	MWD_total_13	[R].LLASDKPVLAEELR.[R]	1xPhospho [S4]; 1xTMTpro [K6]; 1xTMTpro [N-Term]	High	1	3	Q9JI91	Q9JI91 [837-851]	Alpha-actinin-2 OS=Mus musculus OX=10090 GN=Actn2 PE=1 SV=2	Q9JI91 1xPhospho [S840]	0	807.12174	2419.34932	0.55	4.71	0.002746263	0.001588309	-1.729022699	0.000245403	0.038662825
MWD_phospho_438	MWD_total_1	[R].APTPSPVR.[S]	2xPhospho [T3; S5]; 1xTMTpro [N-Term]	High	1	7	A2ASS6	A2ASS6 [297-304]	Titin OS=Mus musculus OX=10090 GN=Ttn PE=1 SV=1	A2ASS6 2xPhospho [T299; S301]	0	430.20551	1288.60229	-0.25	2.41	0.000274345	0.000167274	-1.640098052	0.000285084	0.038662825
MWD_phospho_859	MWD_total_9	[R].KLVIIESDLER.[A]	1xPhospho [S7]; 1xTMTpro [K1]; 1xTMTpro [N-Term]	High	1	5	P58771	P58771 [168-178]	Tropomyosin alpha-1 chain OS=Mus musculus OX=10090 GN=Tpm1 PE=1 SV=1	P58771 1xPhospho [S174]	1	668.38595	2003.14336	-0.03	3.45	0.003882196	0.003416858	-1.136188858	0.000318026	0.038662825
MWD_phospho_600	MWD_total_4	[R].SPTPPSIAAK.[A]	1xPhospho [T/S]; 1xTMTpro [K10]; 1xTMTpro [N-Term]	High	1	49	A2ASS6	A2ASS6 [264-273]	Titin OS=Mus musculus OX=10090 GN=Ttn PE=1 SV=1	A2ASS6 1xPhospho [S264; T266; S269]	0	828.86419	1656.92174	-0.38	3.16	0.000790258	0.000632562	-1.263522262	0.000511216	0.054280557

MYO_phospho_	MYO_total_inde	Annotated Seq	Modifications	Confidence (by	# Proteins	# PSMs	Master Protein	Positions in Ma	Master Protein	Modifications i	# Missed Cleav	m/z [Da] (by Se	Theo. MH+ [Da	DeltaM [ppm] (	XCorr (by Searc	Log2_Mean_M_	Log2_Mean_M	**Linear_Differe**	**Welch_Ttest_**	Welch_Ttest_Corrected_Pvalue
MYO_phospho	MYO_total_25	[K].ASSEGAQG	1xPhospho [S];	High	1	9	Q9JKS4	Q9JKS4 [169-1	LIM domain-bir	Q9JKS4 1xPhos	0	631.65855	1892.96104	0.03	4.3	0.004043853	0.011625322	2.874813631	0.001154884	0.060894164
MYO_phospho	MYO_total_1	[K].KTPSPIEAEI	1xPhospho [S4	High	1	9	A2ASS6	A2ASS6 [12868	Titin OS=Mus m	A2ASS6 1xPhos	1	605.99984	1815.98613	-0.64	2.7	0.000638476	0.00118998	1.863781945	0.00303239	0.066233234
MYO_phospho	MYO_total_1	[K].KLSDHSVEI	1xPhospho [S3	High	1	23	A2ASS6	A2ASS6 [6879-	Titin OS=Mus m	A2ASS6 1xPhos	1	548.0592	2189.21474	0.11	4.84	0.001017701	0.001755352	1.724820878	0.001982884	0.06490132
MYO_phospho	MYO_total_71	[K].TLPQVSPKT	1xPhospho [T9	High	1	6	Q8BWB1	Q8BWB1 [694-	Synaptopodin 2	Q8BWB1 1xPh	1	675.13611	2697.52307	-0.17	5.86	0.016465902	0.028326162	1.7202922	4.88E-05	0.020772792
MYO_phospho	MYO_total_22	[K].SSKLIMELT	1xPhospho [S2	High	1	6	Q9JK37	Q9JK37 [15-27	Myozenin-1 OS	Q9JK37 1xPhos	1	685.03876	2053.10085	0.42	3.78	0.018069124	0.029703891	1.643903185	0.015636026	0.150513801
MYO_phospho	MYO_total_14	[R].TTSTLQREE	1xPhospho [T1	High	1	9	Q62234	Q62234 [207-2	Myomesin-1 OS	Q62234 1xPhos	1	565.29471	2258.15611	0.4	3.33	0.008203568	0.013063426	1.59240773	0.022039386	0.174715138
MYO_phospho	MYO_total_22	[K].SSKLIMELT	1xPhospho [S2	High	1	1	Q9JK37	Q9JK37 [15-27	Myozenin-1 OS	Q9JK37 1xPhos	1	679.70687	2037.10593	0.06	2.94	0.004672305	0.007273757	1.556781142	0.033261404	0.196565657
MYO_phospho	MYO_total_13	[K].MLDAEDIVN	1xPhospho [T1	High	1	14	Q9JI91	Q9JI91 [228-24	Alpha-actinin-2	Q9JI91 1xPhos	0	637.57316	2547.26574	1.99	5.28	0.005047245	0.007727192	1.530972243	0.002749349	0.066233234
MYO_phospho	MYO_total_25	[K].AAQSQLSQ	1xPhospho [S7	High	1	5	Q9JKS4	Q9JKS4 [38-68	LIM domain-bir	Q9JKS4 1xPhos	0	990.49921	3958.97129	0.93	7.6	0.004086563	0.006194518	1.515825882	0.007622666	0.104627235
MYO_phospho	MYO_total_25	[K].SKRPIPISTT	1xPhospho [S1	High	1	8	Q9JKS4	Q9JKS4 [83-10	LIM domain-bir	Q9JKS4 1xPhos	1	739.83263	3695.15935	-6.84	7.99	0.008798067	0.013269286	1.508204778	1.19E-05	0.010119554
MYO_phospho	MYO_total_29	[R].SSSRAEANI	1xPhospho [S];	High	1	49	Q9JIF9	Q9JIF9 [229-24	Myotilin OS=Mu	Q9JIF9 1xPhos	1	813.06686	2437.18518	0.35	5.55	0.122860619	0.184503401	1.501729375	0.004390436	0.079715601
MYO_phospho	MYO_total_31	[R].SVTSPISDF	2xPhospho [T/Y	High	1	24	Q91YE8	Q91YE8 [600-6	Synaptopodin-2	Q91YE8 2xPhos	0	1088.84692	3264.52264	1.1	6.91	0.018601119	0.026625135	1.431372742	0.048461906	0.233853685
MYO_phospho	MYO_total_314	[R].EMEKSFDEI	1xPhospho [S5	High	1	7	Q8K4L3	Q8K4L3 [662-6	Supervillin OS=	Q8K4L3 1xPhos	1	647.08369	2585.31386	-0.36	2.68	0.287062067	0.406387061	1.415676637	0.002879203	0.066233234
MYO_phospho	MYO_total_71	[R].GPARPSLP	2xPhospho [Y/S	High	1	13	Q8BWB1	Q8BWB1 [158-	Synaptopodin 2	Q8BWB1 2xPh	0	1194.93237	3582.7778	1.33	4.66	0.045047634	0.063288177	1.404916793	0.010094129	0.122722339
MYO_phospho	MYO_total_29	[R].SRSSSRAEA	2xPhospho [S];	High	1	11	Q9JIF9	Q9JIF9 [227-24	Myotilin OS=Mu	Q9JIF9 2xPhos	2	690.8269	2760.28465	0.41	5.95	0.013228251	0.018441639	1.394110202	0.001561411	0.06490132
MYO_phospho	MYO_total_31	[R].MQSSVSES	2xPhospho [S];	High	1	20	Q91YE8	Q91YE8 [538-5	Synaptopodin-2	Q91YE8 2xPhos	0	686.27423	2056.80774	0.19	3.78	0.018372874	0.025600155	1.39336691	0.006622042	0.102461043
MYO_phospho	MYO_total_314	[R].KFSLKEFGE	1xPhospho [S3	High	1	2	Q8K4L3	Q8K4L3 [1009-	Supervillin OS=	Q8K4L3 1xPhos	2	1084.24479	3250.71768	0.66	4.86	0.803489833	1.118783019	1.392404698	0.022836493	0.178292255
MYO_phospho	MYO_total_91	[R].LSALGRSPI	1xPhospho [S7	High	1	6	Q62407	Q62407 [388-3	Striated muscle	Q62407 1xPhos	1	447.58365	1340.73707	-0.49	3.24	0.099147504	0.136342102	1.375144065	0.008196647	0.108989787
MYO_phospho	MYO_total_39	[R].KISQTAQTY	1xPhospho [S3	High	1	13	E9PZQ0	E9PZQ0 [2842-	Ryanodine rece	E9PZQ0 1xPhos	1	524.78125	2096.10329	-0.06	2.65	0.098270937	0.134299535	1.366625151	0.022173014	0.174715138
MYO_phospho	MYO_total_25	[K].RPIPISTTAP	1xPhospho [S1	High	1	13	Q9JKS4	Q9JKS4 [85-10	LIM domain-bir	Q9JKS4 1xPhos	0	794.71134	3175.82521	-0.53	7.71	0.037058399	0.050123591	1.352556839	0.008777825	0.113180742
MYO_phospho	MYO_total_1	[R].APTPSPVRS	4xPhospho [T3	High	1	43	A2ASS6	A2ASS6 [297-3	Titin OS=Mus m	A2ASS6 4xPhos	1	701.63025	2102.87987	-1.75	5.93	0.001120502	0.000826565	-1.35561171	0.001727867	0.06490132
MYO_phospho	MYO_total_20	[R].APSYGAGE	1xPhospho [S/Y	High	1	20	P31001	P31001 [79-10	Desmin OS=Mu	P31001 1xPhos	0	1047.18194	3139.53083	0.14	8.48	0.003046851	0.002247037	-1.35594176	0.011099374	0.124283785
MYO_phospho	MYO_total_1	[R].IELSPSMEA	1xPhospho [S6	High	1	4	A2ASS6	A2ASS6 [2075-	Titin OS=Mus m	A2ASS6 1xPhos	0	630.66978	1889.99392	0.46	3.14	0.000626389	0.000461051	-1.35861164	0.015741161	0.150513801
MYO_phospho	MYO_total_8	[K].LEKSIDDLE	1xPhospho [S4	High	1	2	P58771	P58771 [249-2	Tropomyosin al	P58771 1xPhos	1	866.4466	2597.3243	0.37	5.06	0.001467617	0.001078444	-1.36086543	0.026869066	0.195432267
MYO_phospho	MYO_total_1	[R].SLGDMSDE	1xPhospho [S];	High	1	5	A2ASS6	A2ASS6 [33875	Titin OS=Mus m	A2ASS6 1xPhos	0	1019.51051	3056.51046	2.13	8.37	9.23E-05	6.76E-05	-1.36481556	0.029818363	0.196565657
MYO_phospho	MYO_total_1	[R].QQSPSPIRI	3xPhospho [S5	High	1	20	A2ASS6	A2ASS6 [279-2	Titin OS=Mus m	A2ASS6 3xPhos	1	739.66943	2216.99391	-0.08	5.12	0.000603697	0.000439186	-1.37458112	0.001216452	0.060894164
MYO_phospho	MYO_total_369	[R].LGSRPSIQE	2xPhospho [S3	High	1	2	O70373	O70373 [203-2	Xin actin-bindi	O70373 2xPhos	0	758.7121	2274.12154	0.09	3.27	0.383862203	0.278822254	-1.37672728	0.038851946	0.216098077
MYO_phospho	MYO_total_1	[R].APTPSPVR.	1xPhospho [T/S	High	1	5	A2ASS6	A2ASS6 [297-3	Titin OS=Mus m	A2ASS6 1xPhos	0	403.54973	1208.63595	-1.09	2.21	0.0001184	8.59E-05	-1.37782745	0.001018069	0.060894164
MYO_phospho	MYO_total_1	[R].TASPHFTVS	1xPhospho [T/S	High	1	14	A2ASS6	A2ASS6 [812-8	Titin OS=Mus m	A2ASS6 1xPhos	0	588.31747	1762.93846	-0.34	4.3	0.000248307	0.000179797	-1.38103931	0.034969718	0.201305386
MYO_phospho	MYO_total_13	[R].ISSSNPYST	1xPhospho [S/Y	High	1	12	Q9JI91	Q9JI91 [593-60	Alpha-actinin-2	Q9JI91 1xPhos	0	734.01117	2200.0164	1.16	3.84	0.005772927	0.004160499	-1.38755626	0.005436143	0.094411375
MYO_phospho	MYO_total_1	[R].TPSPDYDLY	1xPhospho [S/T	High	1	18	A2ASS6	A2ASS6 [33859	Titin OS=Mus m	A2ASS6 1xPhos	0	646.29368	1936.86893	-1.26	3.74	0.001228876	0.000882471	-1.39253969	0.001037637	0.060894164
MYO_phospho	MYO_total_1	[R].YSSPPAHVI	1xPhospho [S/Y	High	1	143	A2ASS6	A2ASS6 [33931	Titin OS=Mus m	A2ASS6 1xPhos	0	558.63544	1673.89078	0.59	2.62	0.002692094	0.001928542	-1.39592244	0.018013937	0.159686046
MYO_phospho	MYO_total_40	[R].LRLSPSPTS	2xPhospho [S4	High	1	3	P48678	P48678 [387-3	Prelamin-A/C O	P48678 2xPhos	1	569.28339	1705.83587	-0.15	2.96	0.016169474	0.011525076	-1.40298194	0.01102291	0.124283785
MYO_phospho	MYO_total_1	[R].RTPSPDYDI	1xPhospho [S/T	High	1	24	A2ASS6	A2ASS6 [33858	Titin OS=Mus m	A2ASS6 1xPhos	1	698.32735	2092.97004	-1.22	5.24	0.001060172	0.000754796	-1.40458203	0.002657034	0.066233234
MYO_phospho	MYO_total_3	[K].TEGGATVTV	1xPhospho [S/T	High	1	5	Q5SX39	Q5SX39 [64-87	Myosin-4 OS=M	Q5SX39 1xPhos	2	727.97614	3635.84844	0.87	2.8	0.000386792	0.000275277	-1.40510294	0.041164892	0.216324716
MYO_phospho	MYO_total_1	[R].LSEYKSELD	1xPhospho [S6	High	1	1	A2ASS6	A2ASS6 [34004	Titin OS=Mus m	A2ASS6 1xPhos	1	867.78248	2601.33393	-0.41	2.35	0.000125657	8.91E-05	-1.41005608	0.00412356	0.077981102
MYO_phospho	MYO_total_1	[R].QQSPSPIRI	2xPhospho [S];	High	1	14	A2ASS6	A2ASS6 [279-2	Titin OS=Mus m	A2ASS6 2xPhos	1	535.01233	2137.02758	-0.05	2.83	0.000189181	0.000130259	-1.45234235	0.002468773	0.066233234
MYO_phospho	MYO_total_419	[R].KDLFANTVL	1xPhospho [T7	High	1	2	Q8BFZ3	Q8BFZ3 [292-3	Beta-actin-like	Q8BFZ3 1xPhos	1	905.106	2713.32275	-7.1	2.62	1.142847039	0.786579333	-1.45293296	0.014768153	0.146136026
MYO_phospho	MYO_total_1	[R].APTPSPVRS	3xPhospho [T3	High	1	10	A2ASS6	A2ASS6 [297-3	Titin OS=Mus m	A2ASS6 3xPhos	1	674.97614	2022.91354	0.16	5.98	0.000358667	0.000243698	-1.4717652	0.007058504	0.103710431
MYO_phospho	MYO_total_3	[K].TEGGATVTV	1xPhospho [T/S	High	1	10	Q5SX39	Q5SX39 [64-84	Myosin-4 OS=M	Q5SX39 1xPhos	1	975.82369	2925.45606	0.16	3.25	0.000391762	0.00026226	-1.49379277	0.021900741	0.174715138
MYO_phospho	MYO_total_1	[R].RRTPSPDYI	2xPhospho [T3	High	1	42	A2ASS6	A2ASS6 [33857	Titin OS=Mus m	A2ASS6 2xPhos	2	583.01501	2329.03748	0.32	5.86	0.001337188	0.000886165	-1.5089597	0.001324734	0.06263049
MYO_phospho	MYO_total_71	[R].SPSPGELQ	1xPhospho [S];	High	1	13	Q8BWB1	Q8BWB1 [92-1	Synaptopodin 2	Q8BWB1 1xPh	0	980.27041	3918.05846	0.34	9.48	0.022065017	0.014503705	-1.52133655	0.013906709	0.1443245
MYO_phospho	MYO_total_9	[R].VGEPSMSA	1xPhospho [S];	High	1	19	O88990	O88990 [314-3	Alpha-actinin-3	O88990 1xPhos	0	536.90747	1608.70821	-0.22	3.57	0.008266958	0.00538819	-1.53427382	0.015558259	0.150513801
MYO_phospho	MYO_total_31	[R].TAKPFLGSN	1xPhospho [S/T	High	1	21	Q91YE8	Q91YE8 [580-5	Synaptopodin-2	Q91YE8 1xPhos	0	840.08767	2518.24846	0	5.54	0.0334343	0.021082767	-1.5858592	0.048233727	0.233853685
MYO_phospho	MYO_total_1	[K].YTFESDKGL	1xPhospho [T/Y	High	1	4	A2ASS6	A2ASS6 [33527	Titin OS=Mus m	A2ASS6 1xPhos	1	950.46882	3798.79583	15.17	3.23	0.000127212	8.00E-05	-1.5909368	0.01161687	0.126526191
MYO_phospho	MYO_total_1	[R].RRTPSPDYI	1xPhospho [T/S	High	1	13	A2ASS6	A2ASS6 [33857	Titin OS=Mus m	A2ASS6 1xPhos	2	563.02308	2249.07115	-0.29	5.33	0.000351988	0.000216944	-1.62248134	0.002533957	0.066233234
MYO_phospho	MYO_total_71	[R].SPPDSEAYY	1xPhospho [Y/S	High	1	26	Q8BWB1	Q8BWB1 [127-	Synaptopodin 2	Q8BWB1 1xPh	0	875.65643	3499.60339	0.15	6.1	0.061004101	0.037318212	-1.63470053	0.010671508	0.122722339
MYO_phospho	MYO_total_1	[R].APTPSPVR.	2xPhospho [T3	High	1	7	A2ASS6	A2ASS6 [297-3	Titin OS=Mus m	A2ASS6 2xPhos	0	430.20551	1288.60229	-0.25	2.41	0.000274345	0.000167274	-1.64009805	0.000285084	0.038662825
MYO_phospho	MYO_total_445	[R].SSPPKDSEV	1xPhospho [S];	High	1	6	Q9D2N4	Q9D2N4 [362-	Dystrobrevin al	Q9D2N4 1xPho	1	711.6958	2133.07205	0.38	2.92	1.188806435	0.722717462	-1.64491174	0.016613971	0.15367923
MYO_phospho	MYO_total_326	[R].YHGHSMSI	2xPhospho [Y/S	High	1	4	P35486	P35486 [289-3	Pyruvate dehyd	P35486 2xPhos	0	691.6134	2072.82577	-0.06	3.54	0.324827696	0.195538129	-1.66119875	0.029679374	0.196565657
MYO_phospho	MYO_total_10	[R].RAGAEGSS	1xPhospho [S];	High	1	2	P97457	P97457 [9-31]	Myosin regulat	P97457 1xPhos	1	993.12909	2977.37222	0.17	5.58	0.002774514	0.001657033	-1.67438627	0.032363779	0.196565657
MYO_phospho	MYO_total_326	[R].YHGHSMSI	1xPhospho [S/Y	High	1	11	P35486	P35486 [289-3	Pyruvate dehyd	P35486 1xPhos	0	664.95814	1992.85944	0.21	2.15	0.324827696	0.191531907	-1.6959456	0.031186125	0.196565657
MYO_phospho	MYO_total_13	[R].ILASDKPYIL	1xPhospho [S4	High	1	2	Q9JI91	Q9JI91 [837-85	Alpha-actinin-2	Q9JI91 1xPhos	0	807.12174	2419.34933	0.55	4.71	0.002746363	0.001589309	-1.7280237	0.000245403	0.038662825
MYO_phospho	MYO_total_1	[R].VKSPETVKS	1xPhospho [S9	High	1	7	A2ASS6	A2ASS6 [34462	Titin OS=Mus m	A2ASS6 1xPhos	2	548.82719	2192.28718	-0.12	5.84	0.000382913	0.000202077	-1.89488456	0.034643787	0.201305386
MYO_phospho	MYO_total_1	[K].RVKSPEPVT	1xPhospho [S4	High	1	18	A2ASS6	A2ASS6 [34473	Titin OS=Mus m	A2ASS6 1xPhos	2	538.30479	2150.19786	-0.25	3.46	0.000700094	0.000355003	-1.97207907	0.025885656	0.193234155
MYO_phospho	MYO_total_69	[R].TLSDYNIQK	1xPhospho [S1	High	4	2	P62984	P62984 [55-72	Ubiquitin-60S r	P62984 1xPhos	1	705.38942	2818.53596	-0.04	5.94	0.071455557	0.016381672	-4.36192096	0.007504935	0.104627235

TABLE S3. PKCα-regulated or mechanotransduction-relevant phosphoproteins altered by RNAi-sh16

Protein	Fraction	Mouse Phosphosite(s) Detected	Fold Δ (TR/SC)	Direction	Welch_Ttest_ Uncorrected_ p-value	Welch_Ttest_ Corrected_p-value	PKCα Substrate?	Role in Muscle & Mechanotransduction	Relationship to LDB3–PKCα–FLNC axis	References
**Filamin-C (FLNC)**	CYTO	**S2234, S2237**	**1.37**	↑	0.059753789*	0.18*	**Yes** (PKCα prevents calpain cleavage of FLNC)	Main **Z-disc mechanosensor**; stabilizes sarcomeres	Direct substrate of PKCα; interacts physically with LDB3.	[1–3]
**LDB3 (Cypher/ZASP)**	CYTO	S179	**2.77**	↑	0.00014	0.01	**PKCα-binding scaffold**	Z-disc anchoring protein; recruits PKCα and FLNC	Central hub of LDB3–PKCα–FLNC signaling	[2,4,5]
LDB3	CYTO	S98	**1.9**	↑	0.00289	0.049	Scaffold (LIM domain)	Z-disc anchoring protein; recruits PKCα and FLNC	↑ phosphorylation indicates restored PKCα positioning	[2,4,5]
**LDB3 (MYO fraction)**	MYO	S98	**1.51**	↑	0.00001	0.01	Scaffold (LIM domain)	Myofibrillar scaffold state	Shows compartment-specific PKCα rescue	[2]
**FXYD1 (Phospholemman)**	CYTO	S82/S83	**2.49**	↑	0.00015	0.01	**Yes (canonical PKCα substrate)**	Regulates Na /K^{+} pump; modulates Ca^{2} loading	PKCα normalization restores membrane excitability linked to Z-disc tension	[6–7]
**MARCKS**	CYTO	S138/S141/T143	**2.12**	↑	0.00004	0.006	**Yes (classic PKC substrate)**	Actin–membrane tethering	Reorganization of membrane–cytoskeleton on tension after PKCα rescue	[8]
**CKM (Creatine kinase M-type)**	CYTO	S164	**2.68**	↑	0.00014	0.01	**Yes**	Maintains ATP buffering near myofibrils	Local metabolic rescue required for FLNC stability	[9,10]
CKM	CYTO	S372	**1.64**	↑	0.00003	0.006	Yes	Maintains ATP buffering near myofibrils	Reflects PKCα activation	[9,10]
**ATP2A1 (SERCA1)**	CYTO	S547	**2.32**	↑	0.00019	0.01	**PKC-regulated** (via PLN/SERCA)	Ca^{2} reuptake into SR	Improved Ca^{2} cycling reduces FLNC mechanical strain	[11]
**CASQ1 (Calsequestrin-1)**	CYTO	Y57	**1.38**	↑	0.00066	0.021	PKC-regulated indirectly	SR Ca^{2} buffering	Helps re-establish FLNC-compatible Ca^{2} environment	[12]
**SPEG**	MYO	S2322–2333 cluster	**1.21**	↑	0.00017	0.036	PKC-linked remodeling kinase	Myofibrillar remodeling; Z-disc/I-band maintenance	Activated only when Z-disc tension normalizes with restored FLNC	[13]
**Synaptopodin-2 / Synpo2l**	MYO	T702	**1.72**	↑	0.00005	0.021	PKC-responsive	Actin–filamin crosslinking	Mechanotransduction partner of FLNC	[1]
**Desmin**	MYO	S437	**−1.29**	↓	0.00008	0.023	PKC-modulated	IF network linking Z-discs	↓ phosphorylation = reduced cytoskeletal stress after rescue	[14]
**Titin (TTN)**	MYO	T299/S301	**−1.64**	↓	0.00029	0.038	**PKCα phosphorylates titin's Z-disc/I-band regions**	Passive elasticity; mechanotransducer	↓ indicates resolution of titin overstretch stress	[1,15]
Titin	MYO	S264/T266/S269	**−1.26**	↓	0.00080	0.054	Yes	Passive elasticity; mechanotransducer	↓ phosphorylation matches restored Z-disc integrity	[1,15]
**α-Actinin-2 (ACTN2)**	MYO	S840	**−1.73**	↓	0.00025	0.039	Not PKCα substrate, but PKC-modulated	Crosslinks actin to Z-disc; binds LDB3 & FLNC	↓ = reduced stress remodeling at the Z-disc	[4,16]
**Tropomyosin 1 (TPM1)**	MYO	S174	**−1.14**	↓	0.00388	0.038	Regulated by PKC in some contexts	Thin filament regulatory protein	↓ = normalization of thin filament tension	[17]
**GYS1 (Glycogen synthase 1)**	CYTO	S652–S657	**−2.71**	↓	0.00014	0.01	**Yes**	Glycogen metabolism	↓ phosphorylation indicates metabolic normalization	[18]

*For FLNC S2234/S2237, Welch's t-test yielded p = 0.0598; equal-variance t-test yielded p = 0.0457.

? PKCα Substrate assignments are based on published experimental evidence or curated kinase/phosphosite databases (PhosphoSitePlus, UniProt, KEA3). Indirect terms ('PKC-responsive', PKC-modulated') indicate pathway-level association rather than confirmed kinase - substrate activity.

References

1. Reimann L, Wiese H, Leber Y, et al. Myofibrillar Z-discs Are a Protein Phosphorylation Hot Spot with Protein Kinase C (PKCα) Modulating Protein Dynamics. Mol Cell Proteomics. 2017;16(3):346-367. doi:10.1074/mcp.M116.065425.

2. Pathak P, Blech-Hermoni Y, Subedi K, et al. Myopathy associated LDB3 mutation causes Z-disc disassembly and protein aggregation through PKCα and TSC2-mTOR downregulation. Commun Biol. 2021;4(1):355. Published 2021 Mar 19. doi:10.1038/s42003-021-01864-1.

3. Ulbricht A, Eppler FJ, Tapia VE, et al. Cellular mechanotransduction relies on tension-induced and chaperone-assisted autophagy. Curr Biol. 2013;23(5):430-435. doi:10.1016/j.cub.2013.01.064.

4. Zhou Q, Ruiz-Lozano P, Martone ME, Chen J. Cypher, a striated muscle-restricted PDZ and LIM domain-containing protein, binds to alpha-actinin-2 and protein kinase C. J Biol Chem. 1999;274(28):19807-19813. doi:10.1074/jbc.274.28.19807.

5. Cheng H, Zheng M, Peter AK, et al. Selective deletion of long but not short Cypher isoforms leads to late-onset dilated cardiomyopathy. Hum Mol Genet. 2011;20(9):1751-1762. doi:10.1093/hmg/ddr050.

6. Pavlović D, Fuller W, Shattock MJ. The intracellular region of FXYD1 is sufficient to regulate cardiac Na/K ATPase. FASEB J. 2007;21(7):1539-1546. doi:10.1096/fj.06-7269com.

7. Crambert G, Fuzesi M, Garty H, Karlish S, Geering K. Phospholemman (FXYD1) associates with Na,K-ATPase and regulates its transport properties. Proc Natl Acad Sci U S A. 2002;99(17):11476-11481. doi:10.1073/pnas.182267299.

8. Verghese GM, Johnson JD, Vasulka C, Haupt DM, Stumpo DJ, Blackshear PJ. Protein kinase C-mediated phosphorylation and calmodulin binding of recombinant myristoylated alanine-rich C kinase substrate (MARCKS) and MARCKS-related protein. J Biol Chem. 1994;269(12):9361-9367.

9. Keceli G, Gupta A, Sourdon J, et al. Mitochondrial Creatine Kinase Attenuates Pathologic Remodeling in Heart Failure. Circ Res. 2022;130(5):741-759. doi:10.1161/CIRCRESAHA.121.319648.

10. Yang YC, Kao LS. Regulation of sodium-calcium exchanger activity by creatine kinase. Adv Exp Med Biol. 2013;961:163-173. doi:10.1007/978-1-4614-4756-6_14.

11. MacLennan DH, Kranias EG. Phospholamban: a crucial regulator of cardiac contractility. Nat Rev Mol Cell Biol. 2003;4(7):566-577. doi:10.1038/nrm1151.

12. Sanchez EJ, Munske GR, Criswell A, Milting H, Dunker AK, Kang C. Phosphorylation of human calsequestrin: implications for calcium regulation. Mol Cell Biochem. 2011;353(1-2):195-204. doi:10.1007/s11010-011-0787-4.

13. Quan C, Du Q, Li M, et al. A PKB-SPEG signaling nexus links insulin resistance with diabetic cardiomyopathy by regulating calcium homeostasis. Nat Commun. 2020;11(1):2186. Published 2020 May 4. doi:10.1038/s41467-020-16116-9.

14. Krüger M, Linke WA. Titin-based mechanical signalling in myocardium. J Mol Cell Cardiol. 2009;46:490–498. doi:10.1016/j.yjmcc.2009.01.004.

15. Russell B, Solís C. Mechanosignaling pathways alter muscle structure and function by post-translational modification of existing sarcomeric proteins to optimize energy usage. J Muscle Res Cell Motil. 2021;42(2):367-380. doi:10.1007/s10974-021-09596-9.

16. Izaguirre G, Aguirre L, Hu YP, et al. The cytoskeletal/non-muscle isoform of alpha-actinin is phosphorylated on its actin-binding domain by the focal adhesion kinase. J Biol Chem. 2001;276(31):28676-28685. doi:10.1074/jbc.M101678200

17. Heeley DH. Phosphorylation of tropomyosin in striated muscle. J Muscle Res Cell Motil. 2013;34(3-4):233-237. doi:10.1007/s10974-013-9351-z.

18. Marr L, Biswas D, Daly LA, et al. Mechanism of glycogen synthase inactivation and interaction with glycogenin. Nat Commun. 2022;13(1):3372. Published 2022 Jun 11. doi:10.1038/s41467-022-31109-6.

Table S4. Enrichr-KG results for proteins with treatment-responsive phosphosites in sh16–treated *Ldb3*$^{\mathrm{Ala165Val/+}}$ mice muscle

Library	Enriched term	p value	BH-FDR q value	z score	combined score	Overlap genes
MGI_Mammalian_Phenotype_Level_4_2021	impaired skeletal muscle contractility MP:0002841	1.38e-13	3.475e-11	185	5467	CACNB1;DES;CKM;ATP2A1;CASQ1;JPH1;TTN
GO_Biological_Process_2021	sarcomere organization (GO:0045214)	1.16e-09	4.481e-07	144	2973	SYNPO2L;ACTN2;TPM1;CASQ1;TTN
GO_Biological_Process_2021	muscle contraction (GO:0006936)	2.28e-09	4.481e-07	40.7	809.1	DES;ACTN2;FXYD1;TPM1;ANKRD2;ALDOA;TTN
MGI_Mammalian_Phenotype_Level_4_2021	increased variability of skeletal muscle fiber size MP:0009403	6.72e-09	8.466e-07	97.7	1839	DES;LDB3;PYGM;FLNC;TTN
MGI_Mammalian_Phenotype_Level_4_2021	abnormal muscle physiology MP:0002106	1.1e-08	9.203e-07	48.4	886.9	CACNB1;JSRP1;CKM;ATP2A1;CASQ1;TTN
GO_Biological_Process_2021	myofibril assembly (GO:0030239)	1.12e-08	1.464e-06	87.4	1600	SYNPO2L;ACTN2;TPM1;CASQ1;TTN
MGI_Mammalian_Phenotype_Level_4_2021	increased heart weight MP:0002833	5.47e-08	3.445e-06	25.1	419.5	GYS1;LDHA;DES;FXYD1;SPEG;LDB3;SLC25A4
GO_Biological_Process_2021	actomyosin structure organization (GO:0031032)	2.06e-07	2.023e-05	46.7	719	SYNPO2L;ACTN2;TPM1;CASQ1;TTN
MGI_Mammalian_Phenotype_Level_4_2021	abnormal skeletal muscle morphology MP:0000759	2.2e-07	1.108e-05	46	705.9	DES;LDB3;ANKRD2;FLNC;TTN
KEGG_2021_Human	Hypertrophic cardiomyopathy	4.81e-07	2.259e-05	39	567	CACNB1;DES;TPM1;ATP2A1;TTN
GO_Biological_Process_2021	actin-myosin filament sliding (GO:0033275)	5.56e-07	3.183e-05	75.6	1089	DES;ACTN2;TPM1;TTN
KEGG_2021_Human	Dilated cardiomyopathy	6.64e-07	2.259e-05	36.4	517.8	CACNB1;DES;TPM1;ATP2A1;TTN
Reactome_2022	Muscle Contraction R-HSA-397014	1.05e-06	0.0001577	21.5	296.4	CACNB1;DES;ACTN2;FXYD1;TPM1;ATP2A1
KEGG_2021_Human	Calcium signaling pathway	3.4e-06	7.711e-05	17.4	219.7	VDAC2;PHKB;VDAC1;ATP2A1;CASQ1;SLC25A4
KEGG_2021_Human	Necroptosis	7.98e-06	0.0001321	21.4	251.7	HSP90AB1;VDAC2;VDAC1;PYGM;SLC25A4
Reactome_2022	Glycogen Metabolism R-HSA-8982491	9.69e-06	0.0007267	89	1028	GYS1;PHKB;PYGM
KEGG_2021_Human	Arrhythmogenic right ventricular cardiomyopathy	9.71e-06	0.0001321	35.2	405.8	CACNB1;DES;ACTN2;ATP2A1
Reactome_2022	Striated Muscle Contraction R-HSA-390522	2.58e-05	0.001292	62.3	658.1	DES;ACTN2;TPM1
Reactome_2022	Metabolism R-HSA-1430728	0.000114	0.004259	4.59	41.71	ALDH4A1;GYS1;MARCKS;LDHA;HSP90AB1;CKM;CA3;ACSL1;VDAC2;PHKB;VDAC1;PYGM
Reactome_2022	Glycogen Breakdown (Glycogenolysis) R-HSA-70221	0.000267	0.008015	101	829.1	PHKB;PYGM

Enrichr-KG was run using 35 significantly altered protein derived from TMT-based phosphoproteomics (phosphosite abundances normalized to the corresponding total protein). This table reports the complete Enrichr output as exported (all returned terms). p values are nominal enrichment p values and BH-FDR q values are Benjamini–Hochberg adjusted within each library.
